# Ultrasound Irradiation Assisted Synthesis of Luminescent Nano Amide-Functionalized Metal-Organic Frameworks; Application Toward Phenol Derivatives Sensing

**DOI:** 10.3389/fchem.2022.855886

**Published:** 2022-03-14

**Authors:** Xiao-Wei Yan, Maniya Gharib, Leili Esrafili, Su-Juan Wang, Kuan-Guan Liu, Ali Morsali

**Affiliations:** ^1^ College of Food and Bioengineering, Hezhou University, Hezhou, China; ^2^ Department of Chemistry, Faculty of Sciences, Tarbiat Modares University, Tehran, Iran; ^3^ State Key Laboratory of High-efficiency Coal Utilization and Green Chemical Engineering and Ningxia Key Laboratory for Photovoltaic Materials, Ningxia University, Yinchuan, China

**Keywords:** metal-organic framework, nano structure, ultrasound, luminescence, nitroaromatic

## Abstract

Two nano amide-functionalized metal-organic frameworks (MOFs) with molecular formula [Co(oba) (bpta)]·(DMF)_2_ TMU-50 and [Co_2_(oba)_2_ (bpfn)]·(DMF)_2.5_ TMU-51 obtained under ultrasonic method without any surfactants. The only difference between the two selected amide functionalized pillar ligands, N,N′-bis(4-pyridinyl)-terephthalamide (bpta), and N,N′-bis-(4-pyridylformamide)-1,5-naphthalenediamine (bpfn), is related to the naphthyl group, which led to the different luminescence properties of the nano frameworks. In this study, the special ability of the luminescent nano MOFs were investigated to sensitize nitroaromatic compounds. Due to its unique and porous framework, Nano TMU-50 shows a good sensitivity towards nitro phenol by strong fluorescence emission with a detection limit of 2 × 10^–3^ mM^−1^. Both nano MOF structures were characterized via many analyses such as powder X-ray diffraction, Field Emission Scanning Electron Microscopy (FE-SEM), elemental analysis, and FTIR spectroscopy. Moreover, the effect of a number of important parameters including initial reagent concentrations, power of ultrasound, time on morphology, and size of nano structures were examined. According to the fluorescence titration results, the activated nano-TMU-50 detected NP selectively with a quick response.

## Introduction

The spread of pollutants and their harmful effects on the lives of living organisms are some of the most important environmental issues of recent years. We all know that some harmful materials are known as toxic compounds and can have a serious effect on human health and environmental pollution (Kumar et al., 2015). High-precision sensors are needed to detect pollutants, especially with the capability of detecting dangerous amounts of them for the health of living organisms. It should also be possible to identify target molecules in the presence of other toxic molecules (Di Pietrantonio et al., 2012). The preparation of compounds with good potential for specific adsorption of pollutants is important in both environmental issues and analytical sciences (Huang et al., 2011).

Metal-Organic Frameworks (MOFs) (O’Keeffe, 2009; Yin et al., 2015; Cui et al., 2016) consist of the inorganic clusters and organic pillar ligands which provide a possibility for the creation of strong fluorescent materials with high selectivity, immediate-response, high efficiency, and reversibility. Selecting various ligands with appropriate sizes and functional groups controlling the surface area and pore sizes of MOF compounds (Furukawa et al., 2010; Karagiaridi et al., 2015; P.; Li et al., 2016; Zhao et al., 2014). As we know, this feature is unlike prevalent porous materials (Bury et al., 2013; Grünker et al., 2014; Luo et al., 2017). The selectivity for adsorption of analytes and the nature of the high porosity of MOFs can lead to them having a high sensibility. Commonly, the functional sites in the MOF structures can be used as the identification sites to detect different analytes (Hao et al., 2017; Xu et al., 2016; Yao et al., 2012; L.; Zhang et al., 2016). Due to the advantages mentioned above, a major diversity of MOFs has been prepared and investigated and used for the detection of a number of analyte molecules such as biomolecules, volatile organic molecules, and nitro-aromatic compounds. As nanomaterials usually show better application performance than their bulky counterpart (D'Agata et al., 2014; Esrafili et al., 2021; Masoomi et al., 2015; Masoomi and Morsali, 2016; Safarifard and Morsali, 2013), synthesis of nano-sized MOFs is a way to improve their properties in different applications (Esrafili et al., 2018; Gharib et al., 2018; S.-M.; Hu et al., 2012; Masoomi et al., 2014).

Nano MOFs with different morphologies, such as nanorods, nanoplates, nanocubes, and nanospheres, have been produced using solvothermal, precipitation, and microemulsion techniques (Makiura et al., 2010; Deep et al., 2015; Cao et al., 2017; Mai et al., 2017). The ultrasonic technique is a simple, efficient, fast, and Eco-friendly procedure for the construction of nanostructures that are often not obtained through conventional methods (Qiu et al., 2008; Moradi et al., 2019). In this technique, the relatively pure nanocompounds are acquired, which is important for sensing applications (Bigdeli et al., 2020). The ultrasonic method is based on the organization, formation and immediate falling of bubbles in a liquid medium, which leads to the formation of the hot spots up to 5,000 C and a pressure of 500 atm, and progress of the chemical reaction and production of the nanoparticles can be derived, simultaneously. However, the nano-MOFs with various sizes and promising morphologies have been prepared by using ultrasonic irradiation (Esrafili et al., 2017; Jin et al., 2017; X. Z.; Wang et al., 2020). Recently, we synthesized luminescent nanostructured MOF through a sonochemical procedure (Khan and Jhung, 2015; Hao et al., 2018). It was shown that ultrasonic synthesis is an easy and efficient approach for the synthesis of nano MOFs (Sun et al., 2018).

Fluorescence spectroscopy has advantages such as high sensitivity and cost-effectiveness compared to other methods of sensing materials (Kreno et al., 2012; J.; Wang et al., 2019). Many fluorescent MOF structures (Müller-Buschbaum et al., 2015; Buragohain et al., 2016; Hu M.-L. et al., 2020; Liu et al., 2020) have been studied for the sensing and removal of nitro explosive compounds because of security issues and life and environmental hazards (Ahmed et al., 2017; Halder et al., 2017; Ma et al., 2018; Salinas et al., 2012; X.-S.; Wang et al., 2018). Only a few of these compounds (Joarder et al., 2015) display high and fast selectivity sensing activity against phenol derivatives. Due to their electron deficiency property, Nitro explosive compounds tend to react with electron-rich compounds. Therefore, MOFs with electron-rich functional groups have good potential for sensing nitro aromatic compounds such as nitrophenol (NP).

**FIGURE 1:**
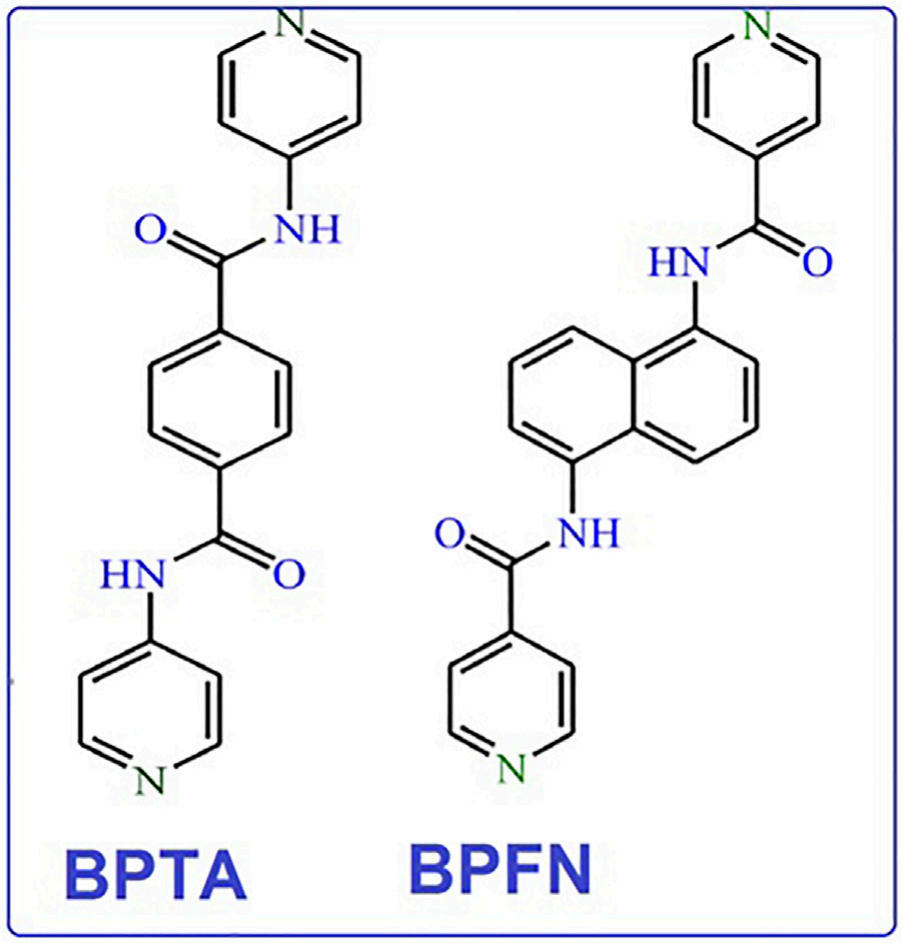
N,Nʹ-bis(4-pyridinyl)-terephthalamide (bpta) and N,Nʹ-bis-(4-pyridylformamide)-1,5-naphthalenediamine (bpfn) linkers.

NP is one of the most common environmental pollutants owing to its widespread use in chemical industries (Germain and Knapp, 2009; M.-L.; Hu et al., 2021). In response, this investigation applied an ultrasonic method to synthesize nano scale MOFs with desirable morphologies and fluorescence properties. This study thus presents the ultrasonic assistant preparation of two new nano luminescent MOFs with an acylamide functional group. The chosen pillar ligands are N,Nʹ-bis(4-pyridinyl)-terephthalamide (bpta) and N,Nʹ-bis-(4-pyridylformamide)-1,5-naphthalenediamine (bpfn), as shown in Figure 1, which led to synthesis of [Co_2_(oba)_2_ (bpta)]·(DMF)_3_ (nano TMU-50): where DMF is N,N-dimethylformamide) and [Co_2_(oba)_2_ (bpfn)]·(DMF)_2_ (nano TMU-51).

The results demonstrate that the presence of the naphthalene analogue affects the pore sizes and induces the acylamide groups to orient towards the cavities in the TMU-50 structure. In addition, in this study, the effect of sonicating time and concentration of reactants on the morphology and size of the nano MOFs is studied. The ultrasonic synthesis of nano TMU-50 and TMU-51 was carried out at different times and concentrations of the initial reagent. Therefore, based on the results, we used the optimal time and concentration to produce nano TMU-50 and TMU-51 at ambient temperature and pressure with ideal sizes and morphologies. More interestingly, we found that both nano plate MOFs, have three-dimensional structure with different sensing properties. The luminescent nano TMU-50 displays high selectivity and fast response time towards 4-nitro phenol (NP) in solution.

## Experimental Section

### Substances and Characterization

Entire chemical materials were commercially prepared. The IR spectra were accomplished on a Nicolet Fourier Transform IR, Nicolet 100 spectrometer *via* the KBr disk method. A computer-controlled PL-STA 1500 device in a Perkin Elmer Pyris one under N_2_ atmosphere with a heating rate of 10 C/min was applied for thermo gravimetric analysis (TGA) of the compounds. Philips diffractometer of X’pert company with monochromated Cu-kα (λ= 1.54056 Å) radiation was used for X-ray powder diffraction (XRD) measurements. Elemental analyses were recorded on a CHNS Thermo Scientific Flash 2000 elemental analyzer. A Misonix Sonicator 3,000 with an adjustable power output (maximum 600 W at 50/60 kHz) was used for sonication. A horn type tube Pyrex reactor was prepared and fitted to the sonicator bar. The size and morphology of the samples were investigated by the field emission scanning electron microscope (FE-SEM) SIGMA ZEISS and TESCAN MIRA (Czech) with gold coating.

### Synthesis of the Ligands

#### Synthesis of Bpta Pillar Ligand

The simple route for the synthesis of amide-containing compounds is the coupling of an acid chloride with an amine group. Note here that the acid chloride-amine reaction is exothermic. Therefore, all organic reactions performed in this study were carried out at a low temperature in the presence of triethylamine (TEA) to capture *in situ* the generated side product HCl. Synthesis of bpta 4-aminopyridine (1.882 g; 20 mmol) and 2.84 ml of TEA (20.4 mmol) were dissolved in 50 ml of dry THF. Then, terephthaloyl chloride (2.030 g; 10 mmol) was added to this solution and heated under reflux for 24 h. The resulting yellow suspension was filtered, dried under ambient conditions, and poured into an aqueous saturated solution of Na_2_CO_3_ (50 ml). The resulting white solid was finally filtered and dried, obtaining the pure ligand bpta in ca. 73% yield.

#### Synthesis of bpfn Pillar Ligand

1, 5-diaminonaphthalene (1.580 g; 10 mmol; for bpfn) were dissolved in 50 ml of dry THF containing 2.84 ml of TEA (20.4 mmol). Then, isonicotinoyl chloride hydrochloride (3.560 g, 20 mmol) was added to these solutions and heated under reflux for 24 h. Both reactions were then treated as above indicated for the synthesis of bpta. The yellowish powders were filtered and dried, obtaining the pure ligands in ca. 87% (bpfn) yields.

### Sonochemical Provision of TMU-50 and 51 Nanoplates

To synthesize nanoTMU-50 and 51, 0.249 g (1 mmol) Co(CH_3_COO)_2_·4H_2_O, 0.258 g (1 mmol) oba and 0.318 g (1 mmol) bpta, and 0.368 g (1 mmol) bpfn were dissolved in 15 ml *N*,*N*′-dimethylformamide (DMF), respectively. The resulting solution was placed in a high-density ultrasonic probe and sonicated with a power output of 305 W for 60 min at room temperature and atmospheric pressure. The separated precipitates were washed with DMF and then dried in air. Pale pink crystals were obtained with a 36% synthesis yield for TMU-50 and purple crystals were obtained with a 38% synthesis yield for TMU-51.

Data of Nano TMU-50. FT-IR (cm^−1^): 1691 (m), 1595 (vs.), 1504 (s), 1395 (vs.), 1331 (m), 1298 (m), 1238 (vs.), 1160 (vs.), 1098 (m), 777 (m), 659 (m), 524 (m). EA on solvent free sample: calcd. (%) for C32 H24 Co N4 O7: C, 60.46; H, 3.81 Co, 9.27; N, 8.82; O, 17.62; found: C, 60; H, 3.5 Co, 9; N, 8.62; O, 17.35.

Data of Nano TMU-51. FT-IR (cm^−1^): 1667 (vs.), 1595(vs.), 1570 (m), 1505 (s), 1386 (vs.), 1235(s), 1158 (vs.), 1089(m), 1065 (m), 1015 (m), 878 (m), 659 (m), 522 (m). EA on solvent free sample: calcd. (%) for C56H46Co2N6O14: C, 58.75; H, 4.05 Co, 10.30; N, 7.34; O, 19.56; found: C, 58.32; H, 4.21 Co, 10.04; N, 8.35; O, 19.08.

The above processes were performed *via* different concentrations (0.01 and 0.05 M) for investigating the effect of initial reagent concentrations on the size and morphology of nanostructure TMU-50 and 51. The initial reagents concentration process was 0.01 M.

### Activation Method

The solvent molecules trapped in the MOF pores can be removed by the solvent exchange method. In here, the synthesized nanoMOFs were placed in 3 ml of CH_3_CN solvent for 3 days, CH_3_CN solution was replaced with the fresh solvent every 24 h. Finally, the CH_3_CN solution was decanted, and the obtained crystals were heated at 100 C for 24 h. The activated sample was then characterized by FT-IR spectroscopy, elemental analysis, and powder X-ray diffraction. The peak at 1665 cm^−1^ in the FT-IR disappeared, showing that DMF molecules were removed after activation. FT-IR data (KBr pellet, cm^−1^) data: selected bands: 3,359 (w), 2929 (w), 1599 (s), 1518 (s), 1382 (m), 1303 (w), 1182 (m), 1022 (w), 841 (w), 783 (w), 535 (w). Anal. calcd for C_46_H_30_N_4_O_12_Co_2_: C, 54.04; H, 3.49; N: 14.54, found: C, 53.92; H, 3.54, N: 14.48.

### Fluorescence Measurements

The Fluorescence properties of NanoTMU-50 and NanoTMU-51 and their daughter compounds were measured in water containing MOFs using a PerkinElmer-LS55 Fluorescence Spectrometer at room temperature. In a typical procedure, 3 mg of an activated MOF was ground down and then immersed in different analyte solutions (3 ml) and after 1 h was tested in the emission mode. For fluorescence measurement in the presence of Nitro aromatics.

### Stern–Volmer Plots

According to the Stern–Volmer equation, (I_0_/I) = K_SV_ [A] + 1. Here, I_0_ is the initial fluorescence intensity of the soaked MOF sample in toluene, I is the fluorescence intensity in the presence of the analyte [A] is the molar concentration of the analyte, and K_SV_ is the Stern–Volmer constant (M^−1^). For the quenching constant extraction, the emission intensity of MOFs was recorded by suspending them into different concentrations of analyte solutions in water, in the same manner, described in the Fluorescence measurement section.

## Results and Discussion

**FIGURE 2:**
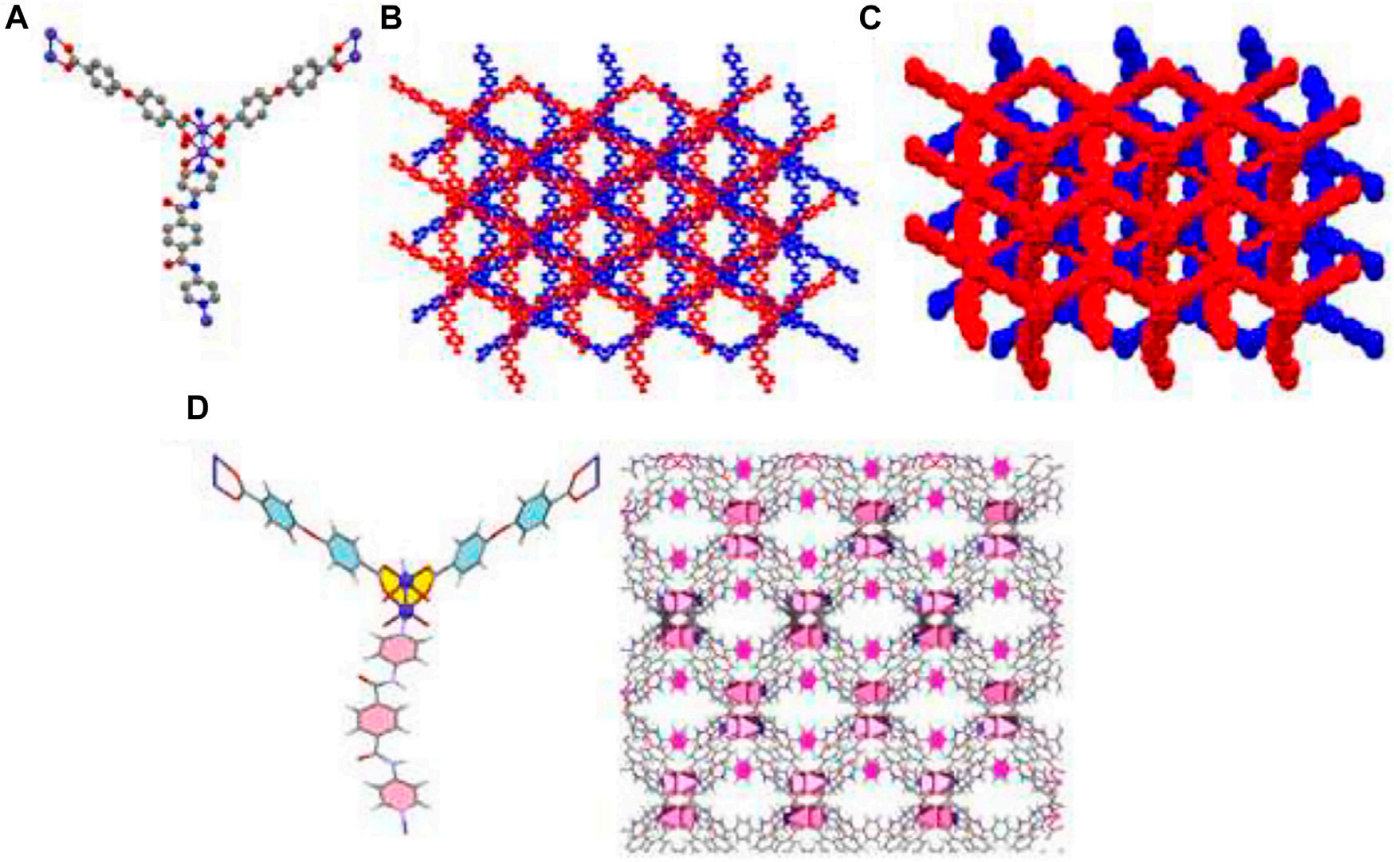
Molecular diagram of TMU-50 **(A)**, showing 3D structure **(B)**, the two-fold interpenetration of TMU-50 **(C)**, and representation of the arrangement of bpta pillar ligands inside the channel walls (with pore scale of 12.84Å). The benzene are colored pink **(D)**.

Due to X-ray data, the crystal system of the compound TMU-50 is monoclinic with the P2_1_/n space group. TMU-50 [Co(oba) (bpta)]•(DMF)_2_, (H_2_oba: 4,4′-oxydibenzoic acid; bpta = N,Nʹ-bis(4-pyridinyl)-terephthalamide was created based on the reaction between Co(NO_3_)_2_, H_2_oba ligand and bpta (amid-based pillar ligand) based on solvothermal method at 120 °C for 24 h. X-ray crystallography shows that this structure includes a binuclear unit of Co (Co1 and Co2). TMU-50 was displayed a paddle-wheel structure. The environmental geometry of this compound is octahedral with six coordination atoms. One nitrogen atom from pyridyl group of bpta pillar ligand, four various coordination modes oxygen atom from oba ligands which created Co-O bands, and one Co-Co band provided TMU-50 coordination environment. The 2D sheets of Co(NO_3_)_2_ and H_2_oba ligand are connected through the linear bpta ligands. According to Figure 2, which is the result of TMU-50 X-ray structural determination, the Co1 atom with octahedral environment coordinated to one nitrogen atom (N1) through bpta pillar ligand, four carboxylate oxygen atoms (O1, O3, O5, and O7) through four oba ligands and the Co2. The coordinated atom to Co2 consists of four oxygen atoms (O2,O4, O6, O8) from four carboxylate groups of oba ligands, one nitrogen atom (N2) through bpta pillar ligand, and Co1, in octahedral mode (Figure 2). As demonstrated in Figure 2B, TMU-50 has two-fold interpenetration. The topology of TMU-50 showed that the structure consists of a 3D framework with V2Ti5ScO2N2C2. The Point symbol for the net is uninodal and 6-connected with point symbol {4^4.6^10.8}.

**FIGURE 3:**
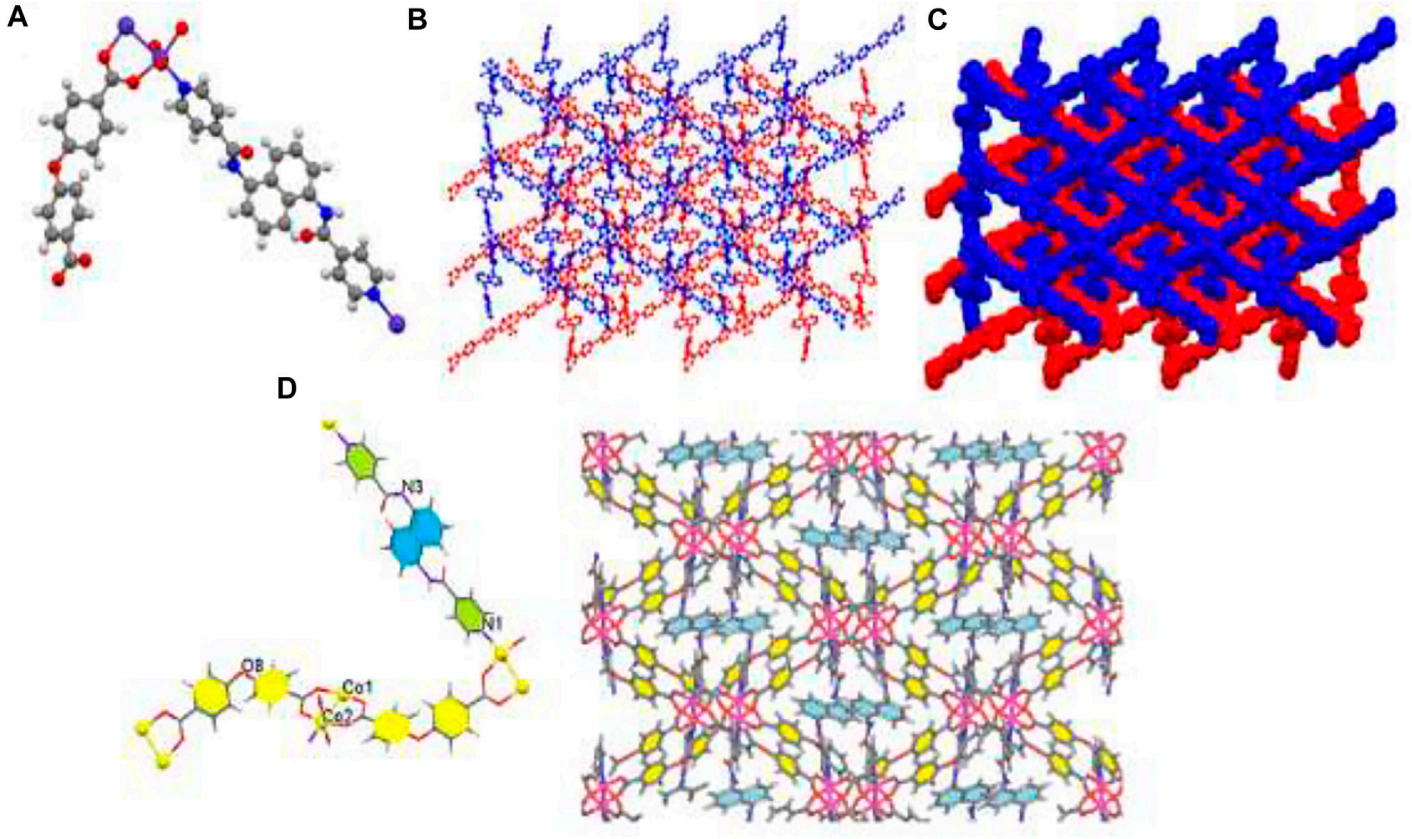
Molecular diagram of TMU-51 **(A)**, Scheme of 3D structure **(B)**, the two-fold interpenetration of TMU-51 **(C)**, and representation of the arrangement of bpfn pillar ligands inside the channel walls (with pore scale of 14.83 Å). The benzene are colored yellow **(D)**.

X-ray crystallography data revealed that TMU-51 structure was crystallized in the orthorhombic system *via* Pna2_1_ space group. Single crystals TMU-51 was prepared through the solvothermal reaction between Co(NO_3_)_2_, bpfn pillar ligand, and the H_2_oba ligand at 120°C for 24 h. The TMU-51 as a 2-fold interpenetrated network contains a binuclear unit of Co (Co1 and Co2). The distorted octahedral coordination environment of Co1-Co2 is surrounded by six atoms consisting of one pyridyl N atom from bpfn pillar ligand and four various O atoms of carboxylate groups through three oba ligands (Figure 3). TMU-51 exhibits a 2-fold interpenetrated network with 1D pore channels of ≈14.83 Å functionalized with acylamide groups. In addition, the Co1 atom is connected to four carboxylate oxygen atoms (O1, O4, O7, O10) from oba ligands, one nitrogen atom (N1) of bpfn, and Co2. In this report, the nano plate of TMU-50 and TMU-51 were synthesized using ultrasonic irradiation with various concentrations (0.01 and 0.05 M) of the reagents. The topology of the TMU-51 net is mab in valence-bonded MOFs cluster representation. The structure consists of a 3D framework with CrVTi2Sc4O2N2C2. It is noteworthy that, replacing benzene core with the naphthalene core increases the contribution percentages of C−H···π and π·π interactions. As depicted in panel d of Figures 2, 3, the bpta and bpfn pillar ligands are arranged inside the channel walls of TMU-50 and TMU-51, respectively.

**FIGURE 4:**
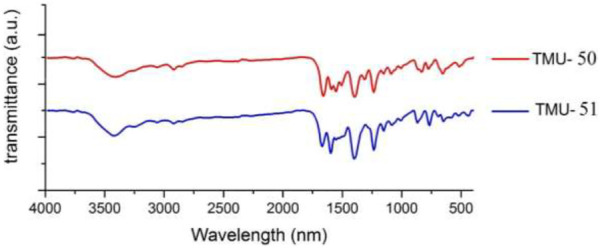
IR spectroscopy of the TMU-50 (red) and TMU-51 (blue).

IR spectra of nanoMOFs prepared using the ultrasonic procedure are consistent with the bulk MOFs synthesized by solvothermal procedure (Figure 4). In the IR spectrum of TMU-50 and TMU-51, the absorption at 3,327 cm^−1^ is assigned to the amide NH absorption stretch. The carboxylate group showed symmetric and asymmetric vibrations of two powerful bands at 1640 and 1690 cm^−1^, respectively. The BET measurement showed that TMU-51 is nonporous toward N_2_ but the TMU-50 structure is porous with a surface area of 753 m^2^/g.

**FIGURE 5:**
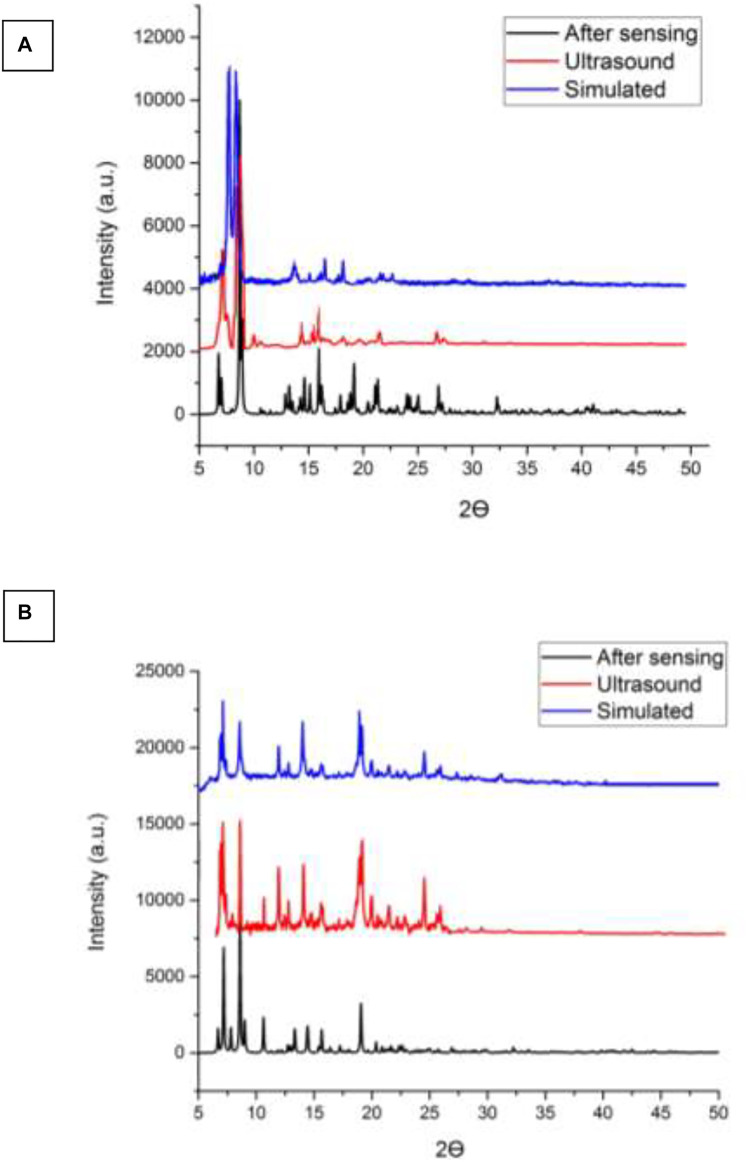
**(A)** XRD patterns of the simulated pattern using by the single crystal data, as synthesized and nanostructure of compound **(A)** TMU-50 **(B)** TMU-51 prepared by the sonochemical process.

According to Figure 5, there is a good agreement between the simulated XRD pattern of the single crystal X-ray data of TMU-50 and TMU-51, the XRD pattern of the prepared single crystals, and nanocompounds synthesized by the ultrasonic method. Therefore, this shows that the nanoMOFs prepared by the ultrasonic process and solvothermal method are completely the same. A little bit of broadening of the peaks in the PXRD for TMU-50 and TMU-51prepared by ultrasonic method shows that the particles are in nanometer scales. There is just a slight difference in Powder X-ray diffraction between the simulated patterns and the as-synthesized materials. The ultrasonic synthesis gives rise to stronger growth in some crystallographic planes so the only difference in those peaks is the intensity of peaks. This points to TMU-51 as being a flexible framework and helps to explain why this framework is nonporous to N_2_ but adsorbs aromatic guests. Guests that give rise to stronger host−guest interactions because aromatic molecules are capable of inflating such flexible frameworks again.

**FIGURE 6:**
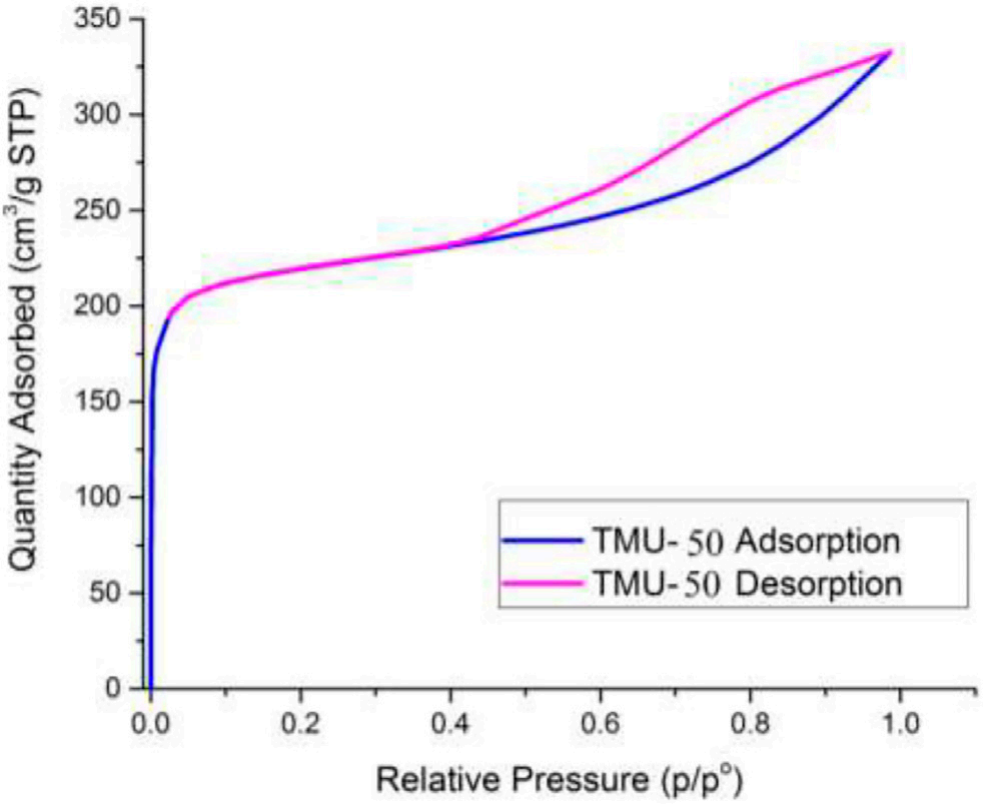
Nitrogen adsorption-desorption isotherms at 77 K. for Nano-TMU-50.

Nitrogen sorption of nano MOFs (TMU-50 and TMU-51) were studied at 77 K (Figure 6). The surface area of TMU-50 is 820 m^2^/g, which was estimated by using BET (Brunauer−Emmett−Teller) theory with a microporous structure. According to the results of the BET analysis, TMU-51 is nonporous toward N_2_. This non-porosity may be because of structural changes during absorption or when subjected to cryogenic temperature under vacuum, which reduces or prevents access to porosity.

**FIGURE 7:**
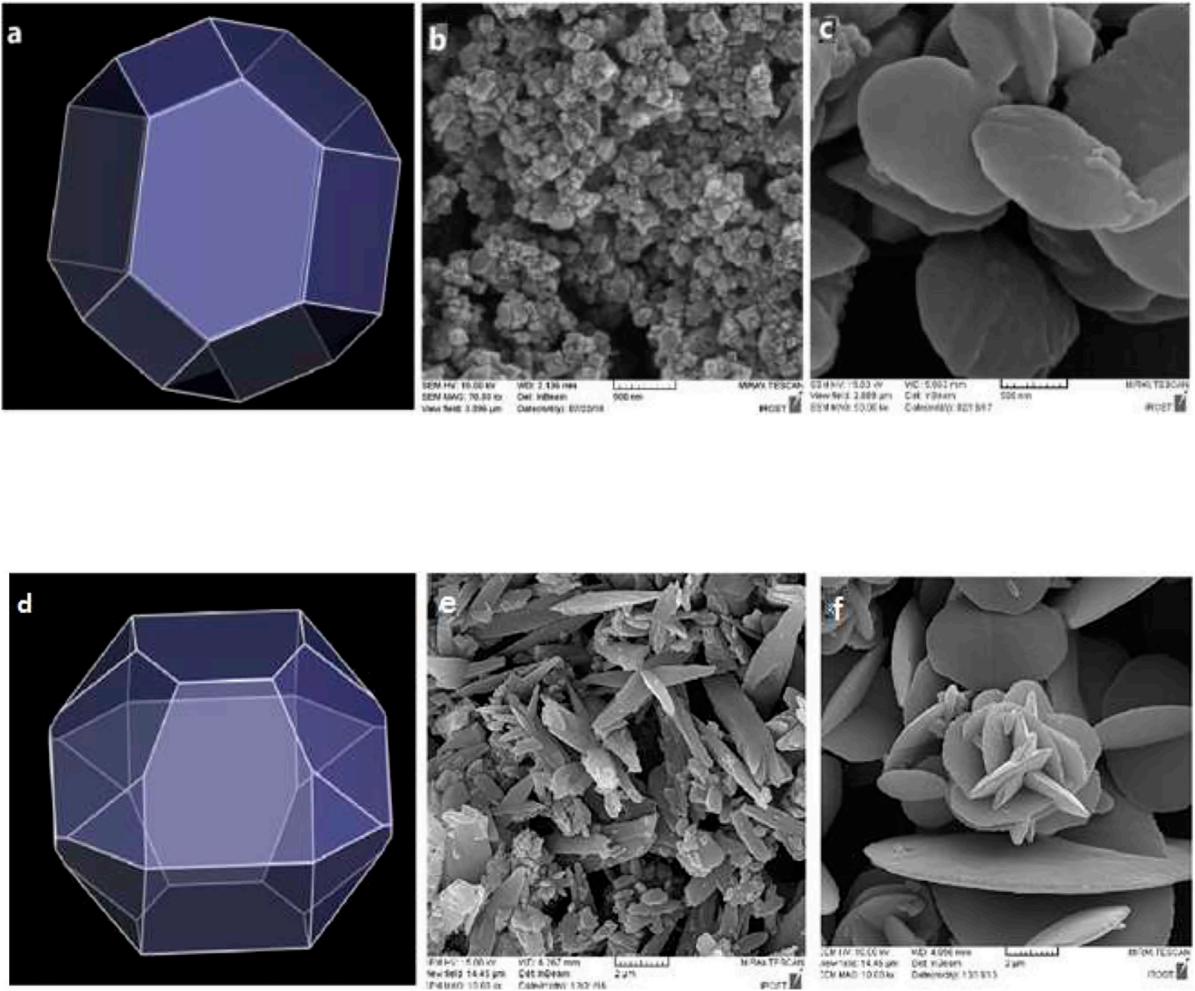
Predicted morphology of **(A)** TMU-50 and **(D)** TMU-51 in DMF by BFDH method. FE-SEM images of TMU-50 **(B)**: 0.01 M **(C)**: 0.05 M (particle size 10–50 nm), and TMU-51 **(E)**: 0.01 M **(F)**: 0.05 M and by using 1 h sonication time (particle size 100–500 nm). Luminescence properties and Sensing of TMU-50 and 51.

The effect of concentration change on the feature of TMU-50 and TMU-51 nano structures was investigated. The nanoparticle features were investigated by two different current concentrations. According to Figure 7, the SEM images show that the samples with higher concentrations to 0.05 M created the larger particle sizes (micro-sized particles) with the agglomerated and fine nano-plate morphology. The outcome display that both size and morphology of TMU- 50 and TMU-51 nanostructures depend on time and concentration. By increasing time to 2 h for the concentration 0.01 M, all nano structures were agglomerated. Therefore, the optimal time for the ideal morphology is 1 h.

MOFs containing π-conjugated ligands show good potential for luminescence properties and therefore the proper candidates for sensing applications (Allendorf et al., 2009). Both TMU-50 and TMU-51 are expected to show good photoluminescence (PL) properties because of the presence of π-electron rich conjugated portions. To investigate the accurate sensing ability of nano TMU-50 and TMU-51 towards analytes of nitroaromatic (NAC) fluorescence, the titration process was carried out by the addition of analytes to TMU-50 and TMU-51 nanoplates dispersed in ethanol. The fluorescence emission spectrum of the nano TMU-50 and TMU-51 compounds displays an emission maximum at 400 nm and 320 nm upon excitation at 310 nm for nano TMU-50 and TMU-51, respectively, that is ascribed to the π-π* transition of the aromatic rings (Z.-Q. Li et al., 2008). The photoluminescence behavior of TMU-50 and TMU-51 in usual organic solvents (ethanol, methanol, dichloromethane, acetonitrile, DMF, chloroform) was also studied. The fluorescence studies were carried out in a suspension of 2 mg of reported MOFs in 2 ml of a number of organic solvents. Ethanol suspension of nano structures displayed the most powerful emission band at 380 nm–430 nm when excited at 310 and 320 nm at room temperature for structures. So here, ethanol is an appropriate solvent for the fluorescent experiments.

Amide forms two hydrogen bonds with the oxygen atoms of nitroaromatic and hydroxide (Nagarkar et al., 2013). Nano TMU-50 functionalized with the amide group has a good potential to interact with phenol-substituted aromatic rings. TMU-50 and TMU-51 nanoplates were used for the discovery of nitroaromatic detecting via a luminescence quenching process. The samples of nitrobenzene, 4-nitrophenol, 4-nitroaniline, and 2,4,6-trinitrophenol, 4-methylphenol, phenol, and 1,3-dihydroxybenzene in water (0.2 M) were added into 2 ml of ethanol in which nano-TMU-50 and nano-TMU-51 were dispersed.

In compound nano-TMU-50, the best interaction bonds occur between 4-nitrophenol (NP) and acylamide groups because of the pore size of the nano-TMU-50 which are appropriate toward NP analytes (Supplementary Figures S1–S6). Nitroaromatics are ready to accept electrons excited from the fluorophore compound due to having an unoccupied π* orbital at low energy levels, so they can prevent the fluorescence emission of the fluorophore MOFs (Wu et al., 2015). While, in nano-TMU-51 structure, the amide NH groups of the ligand bpfn are not mainly toward the pores so there is not an effective interaction between analytes and MOF (Supplementary Figures S7–S12). According to many reports, nitroaromatics can quench the emission of the fluorescent compounds via π-interactions including C−H···π and π···π stacking interactions (Dong et al., 2018). The nature of the substitutions of the nitroaromatic rings and the energy level of the Frontier orbitals have an important effect on the level of LUMO and HOMO energies. Nitropehnol and trinirophenol have the highest level of LUMO and aminophenol has the lowest level of HOMO energies due to the existence of the acceptor and donor groups in the nitroaromatic rings. So nitrophenol and trinitrophenol compounds as the electron acceptors are the proper options for our sensing studies.

**FIGURE 8:**
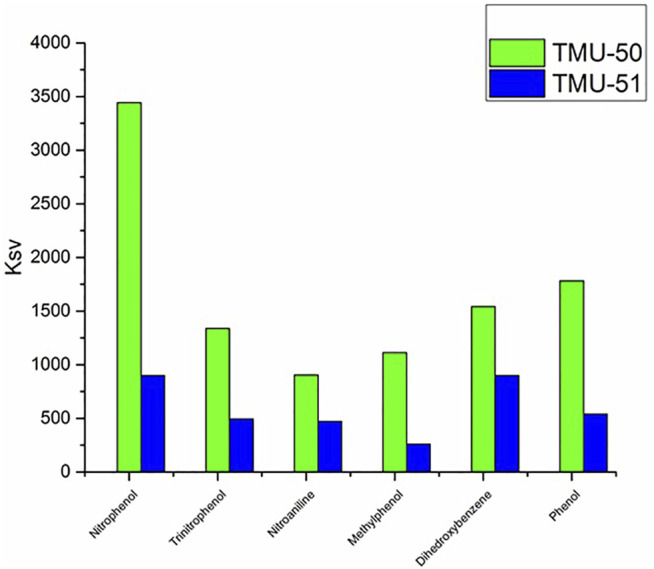
Comparison graph between TMU–50 and TMU-51 in different NACs sensing.

The use of small amounts of nitroaromatic analytes in the solution containing the TMU-50 and TMU-51 leads to an obvious reduction in the fluorescence emission intensity of the nanoMOFs. Among them, nitrophenol has the most fluorescence quenching effect for TMU-50 nano plates. The order of quenching efficiency is nitrophenol > trinitrophenol >4-nitroaniline > methylphenol > dihydroxybenzene (Figure 8) Here, the quenching effect or efficiency is compared with the K_sv_ value of TMU structures towards the nitroaromatic compounds. The fluorescence quenching efficiency is obtained by [(I_0_-I)/I_0_] × 100%, where I_0_ is the initial fluorescence intensity of acetonitrile solution of the MOF sample and I is the fluorescence intensity belongs to the solution containing nitroaromatic molecules (Bagheri et al., 2017). Generally, a more sensitive system will have a steeper slope and, as a result, a higher Ksv value. The Stern–Volmer equation does not contain a variable for the fluorophore concentration and suggests that fluorescence quenching is independent of the fluorophore concentration. The amounts of the K_SV_ for TMU-50 nanoplates against nitroaromatic compounds show that there is a highly selective sensing activity to the nitrophenol compound (Supplementary Figure S13).

The higher quenching effect of the nitroaromatic compounds is attributed to the high electron-withdrawing of the substitute group–NO_2_. The linear correlation coefficient (R) in the Ksv curve indicates that the quenching effect of nitroaromatic compounds on the fluorescence of TMU-50 and 51 is consistent with the Stern–Volmer mode well (Supplementary Figure S14). According to the results, nano MOF TMU-50 can detect nitro aromatic compounds sensitively, so this method is a potential approach for sensing nitroaromatic explosives quickly, easily, and in an environmentally friendly manner.

Decoration of pore-walls and the surface of the porous materials with specific functional groups is a very practical strategy for achieving high fluorescence efficiency. A comparison of the detection limit (LOD) of TMU-50 with reported MOFs with different active sites showed that this structure has comparable detection efficiency towards Nitroaromatic compounds. As an example, Table 1 compares the LOD of reported MOFs and TMU-50 towards nitrophenol.

**TABLE 1:** Comparison of Detection Limit (LOD) of several reported MOFs in nitrophenol sensing.

Compound name	Ligand	Reported LOD	References
In(III)-MOF	H_2_DOBDC (2,5-dihydroxyterephthalic acid)	66 ± 8 ppb	Sharma et al. (2019)
Zn(II)- MOF	H4TCPE (1,1,2,2-tetra(4-carboxylphenyl)ethylene)	36.15 × 10^–9 m^	Zhang X et al. (2019)
Zn(II)-MOF	H_4_TCPP (2,3,5,6-tetrakis (4-carboxyphenyl)pyrazine)	0.66 ppm	Hu Y.-J. et al., 2020
Eu(III)-MOF	HINO (isonicotinic acid N-oxide)	0.08 × 10^–3 m^	Zhang X.-J. et al. (2019)
TMU-50	bpta	0.01 mM	This work

## Conclusion

Two nano plate MOFs including π-conjugated amide functionalized ligands and cobalt metal ion *having* fluorescence properties were synthesized by ultrasonic method [Co(oba) (bpta)]·(DMF)_2_ (TMU-50) and [Co_2_(oba)_2_ (bpfn)]·(DMF)_2.5_ (TMU-51). The effect of the time and concentration parameters on the size and morphology of the prepared nano-MOFs was studied. The results show that nanoparticles agglomerate with increasing time and concentrations. Moreover, nano particles with the best shape and plate morphology were produced by increasing power. According to fluorescence titration results, the activated nano-TMU-50 detected NP with high selectivity and quick response. The detection limit of TMU-50 nano plates for detection of NP was 2 × 10^–5^ M, which is comparable to the amounts of the reported MOF–based fluorescence sensors. Moreover, the enhanced selectivity of nano TMU-50 for sensing NP is due to the electrostatic interactions between the functional group of amide in ligand and the hydroxyl group of NP.

## Data Availability

The original contributions presented in the study are included in the article/Supplementary Material, further inquiries can be directed to the corresponding authors.
