# Supplementary material for: Ultrasound Irradiation Assisted Synthesis of Luminescent Nano Amide-Functionalized Metal-Organic Frameworks; Application Toward Phenol Derivatives Sensing
Source: Front Chem. 2022 Mar 14;10:855886. doi: 10.3389/fchem.2022.855886 (PMC8967136; doi:10.3389/fchem.2022.855886)
Supplement: Supplementary file 1 [file DataSheet1.docx]

**Supporting Information**

**Ultrasounic Assisted Synthesis of Luminescent Nano Amide-Functionalized Metal-Organic Frameworks; Precise Sensors for Phenol derivatives**

Xiao-Wei Yan^a, †^, Maniya Gharib^b, †^, Leili Esrafili^b, †^, Su-Juan Wang^a^*, Kuan-Guan Liu^c^, Ali Morsali^b^*

^a^College of Food and Bioengineering, Hezhou University, No. 18 West Ring Road, Hezhou, Guangxi 542899, P. R. China

^b^Department of Chemistry, Faculty of Sciences, Tarbiat Modares University, Tehran 14115-175, Iran

^c^State Key Laboratory of High-efficiency Coal Utilization and Green Chemical Engineering and Ningxia Key Laboratory for Photovoltaic Materials, Ningxia University, Yinchuan, 750021, PR China

***Corresponding Author:** [2625657354@qq.com](mailto:2625657354@qq.com) （Su-Juan Wang), [morsali_a@modares.ac.ir](mailto:morsali_a@modares.ac.ir) (Ali Morsali)

^†^These authors contribute equally to the work.

1. **Substances and Characterization**

Entire chemical materials were commercially prepared. The IR spectra were accomplished on a Nicolet Fourier Transform IR, Nicolet 100 spectrometer *via* the KBr disk method. Computer-controlled PL-STA 1500 device in a Perkin Elmer Pyris 1 under N_2_ atmosphere with heating rate of 10 °C/min was applied for thermo gravimetric analysis (TGA) of the compounds. Philips diffractometer of X’pert company with monochromated Cu-kα (λ= 1.54056 Å) radiation was used for X-ray powder diffraction (XRD) measurements. Elemental analyses were recorded on a CHNS Thermo Scientific Flash 2000 elemental analyzer. A Misonix Sonicator 3000 with an adjustable power output (maximum 600W at 50/60 kHz) was used for sonication. A horn type tube Pyrex reactor was prepared and fitted to the sonicator bar. The size and morphology of the samples was investigated by the field emission scanning electron microscope (FE-SEM) SIGMA ZEISS and TESCAN MIRA (Czech) with gold coating.

**2. Synthesis of the ligands**

**2.1. Synthesis of bpta pillar ligand**

The simple route for the synthesis of amide-containing compounds is the coupling of an acid chloride with an amine group. Note here that the acid chloride-amine reaction is exothermic. Therefore, all organic reactions performed in this study were carried out at low temperature in the presence of triethylamine (TEA) to capture in situ the generated side product HCl. Synthesis of bpta 4-aminopyridine (1.882 g; 20 mmol) and 2.84 ml of TEA (20.4 mmol) were dissolved in 50 ml of dry THF. Then, terephthaloyl chloride (2.030 g; 10 mmol) was added into this solution and heated under reflux for 24 h. The resulting yellow suspension was filtered, dried under ambient conditions, and poured into an aqueous saturated solution of Na_2_CO_3_ (50 ml). The resulting white solid was finally filtered and dried, obtaining the pure ligand bpta in ca. 73 % yield.

**2.2. Synthesis of bpfn** **pillar ligand**

1, 5-diaminonaphthalene (1.580 g; 10 mmol; for bpfn) were dissolved in 50 ml of dry THF containing 2.84 ml of TEA (20.4 mmol). Then, isonicotinoyl chloride hydrochloride (3.560 g, 20 mmol) was added into these solutions and heated under reflux for 24 h. Both reactions were then treated as above indicated for the synthesis of bpta. The yellowish powders were filtered and dried, obtaining the pure ligands in ca. 87 % (bpfn) yields.

**2.3. Activation Method**

The solvent molecules trapped in the MOF pores can be removed by solvent exchange method. In here, the synthesized nanoMOFs were placed in 3 mL of CH_3_CN solvent for 3 days, CH_3_CN solution was replaced with the fresh solvent every 24 h. Finally, the CH_3_CN solution was decanted, and the obtained crystals were heated at 100 °C for 24 h. afterwards, the activated sample was characterized by FT-IR spectroscopy, elemental analysis and powder X-ray diffraction. The peak at 1665 cm^–1^ in the FT-IR was disappeared, showing that DMF molecules were removed after activation. **Data of activated MOFs:** FT-IR data (KBr pellet, cm^-1^) data: selected bands: 3359 (w), 2929 (w), 1599 (s), 1518 (s), 1382 (m), 1303 (w), 1182 (m), 1022 (w), 841 (w), 783 (w), 535 (w). Anal. calcd for C_46_H_30_N_4_O_12_Co_2_: C, 54.04; H, 3.49; N: 14.54, found: C, 53.92; H, 3.54, N: 14.48.

**2.4. Fluorescence Measurements**

The Fluorescence properties of NanoTMU-50 and NanoTMU-51 and their daughter compounds were investigated in water at room temperature. 3 mg of an activated MOF was grinded down, and then immersed in different analyte solutions (3 ml) and after 1 hour was tested in the emission mode. For fluorescence measurement in the presence of Nitro aromatics.

**2.5. Stern-Volmer Plots**

According to the Stern-Volmer equation, (I_0_/I)= K_SV_ [A] + 1,Where here, I_0_  is the initial fluorescence intensity of soaked MOF sample in toluene, I is the fluorescence intensity in the presence of analyte, [A] is the molar concentration of analyte, and K_SV_ is the Stern-Volmer constant (M^-1^). For the quenching constant extraction, emission intensity of MOFs was recorded by suspending them into different concentrations of analyte solutions in water, upon the same manner described in Fluorescence measurement section.

**Data of Nano TMU-50**. FT-IR (cm^−1^): 1691 (m), 1595 (vs), 1504 (s), 1395 (vs), 1331 (m), 1298 (m), 1238 (vs), 1160 (vs), 1098 (m), 777 (m), 659 (m), 524 (m). EA on solvent free sample: calcd. (%) for C32 H24 Co N4 O7: C, 60.46; H, 3.81 Co, 9.27; N, 8.82; O, 17.62; found: C, 60; H, 3.5 Co, 9; N, 8.62; O, 17.35.

**Data of Nano TMU-51**. FT-IR (cm^−1^): 1667 (vs), 1595(vs), 1570 (m), 1505 (s), 1386 (vs), 1235(s), 1158 (vs), 1089(m), 1065 (m), 1015 (m), 878 (m), 659 (m), 522 (m). EA on solvent free sample: calcd. (%) for C56H46Co2N6O14: C, 58.75; H, 4.05 Co, 10.30; N, 7.34; O, 19.56; found: C, 58.32; H, 4.21 Co, 10.04; N, 8.35; O, 19.08.


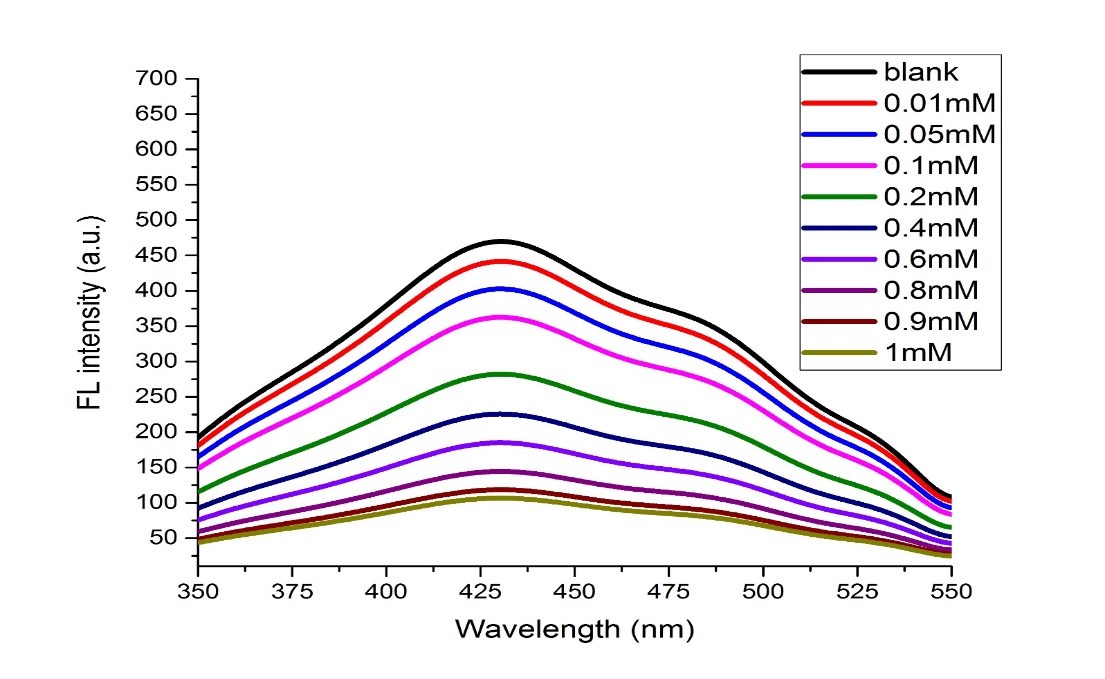


Figure **S1**. Fluorescence emission spectra of **TMU-50** dispersed in water solution at different concentrations of **4-Nitrophenol,** excited at 310 nm.


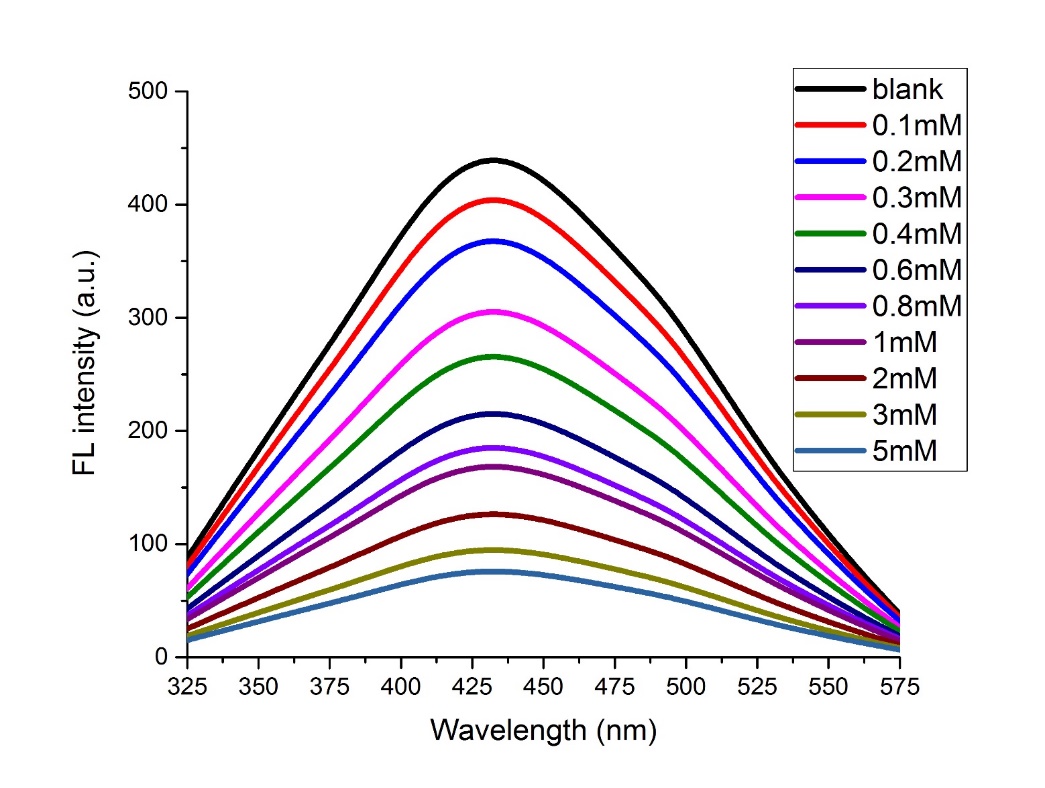


Figure **S2**. Fluorescence emission spectra of **TMU-50** dispersed in water solution at different concentrations of **2, 4, 6-trinitrophenol,** excited at 310 nm.


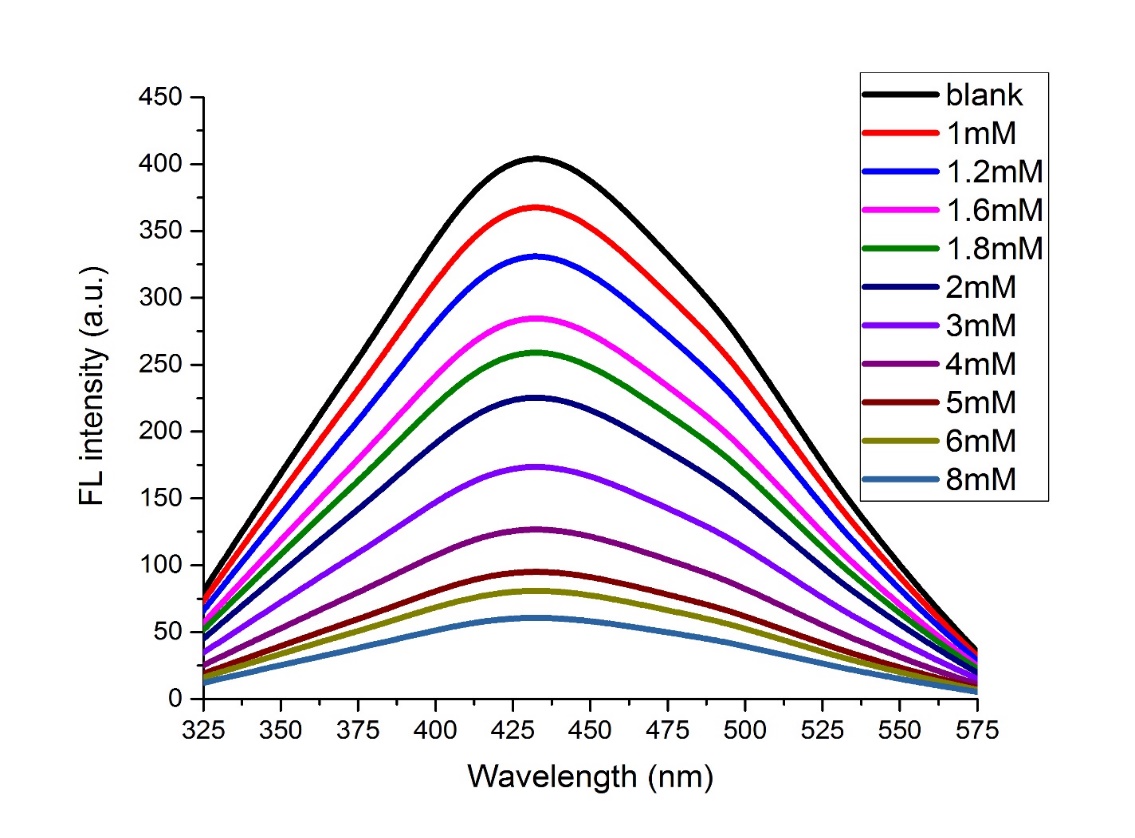


Figure **S3**. Fluorescence emission spectra of **TMU-50** dispersed in water solution at different concentrations of **4-Nitroaniline,** excited at 310 nm.


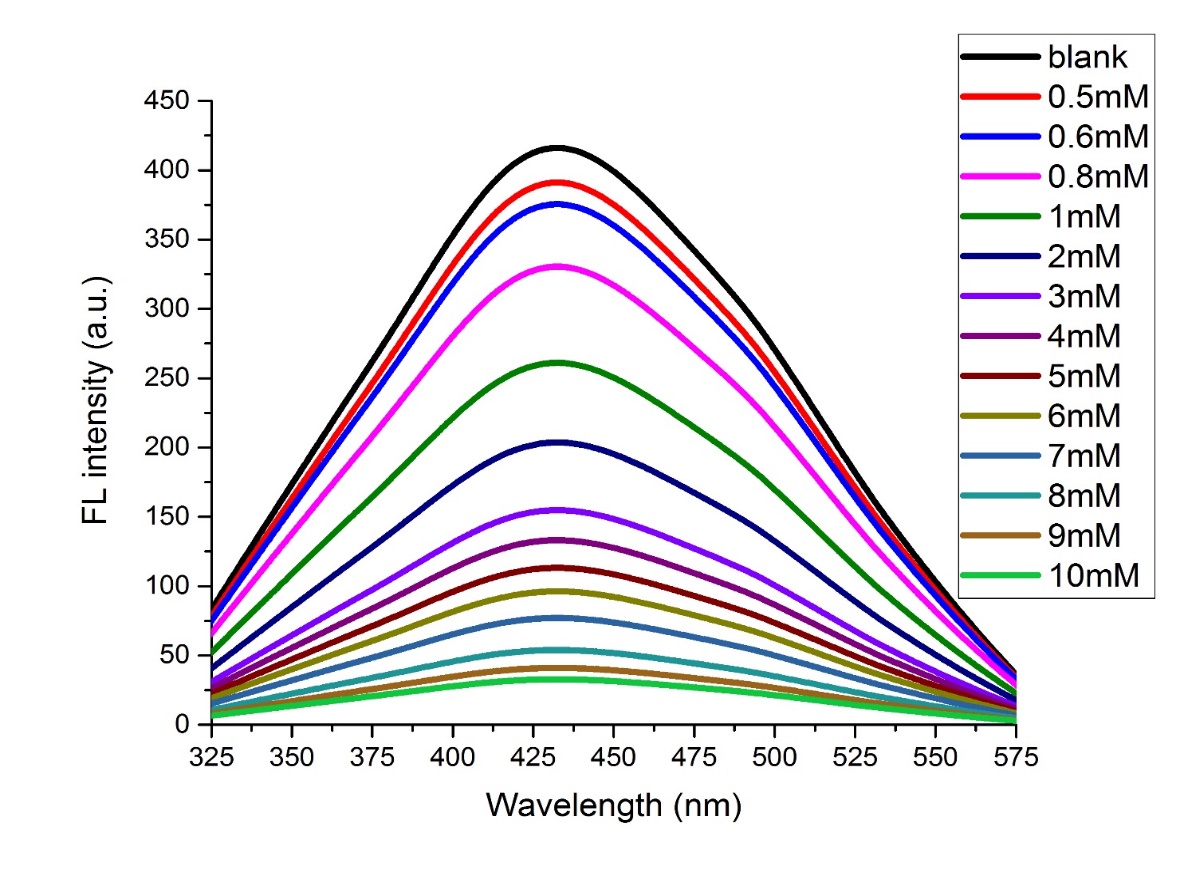


Figure **S4**. Fluorescence emission spectra of **TMU-50** dispersed in water solution at different concentrations of **4-methylphenol,** excited at 310 nm.


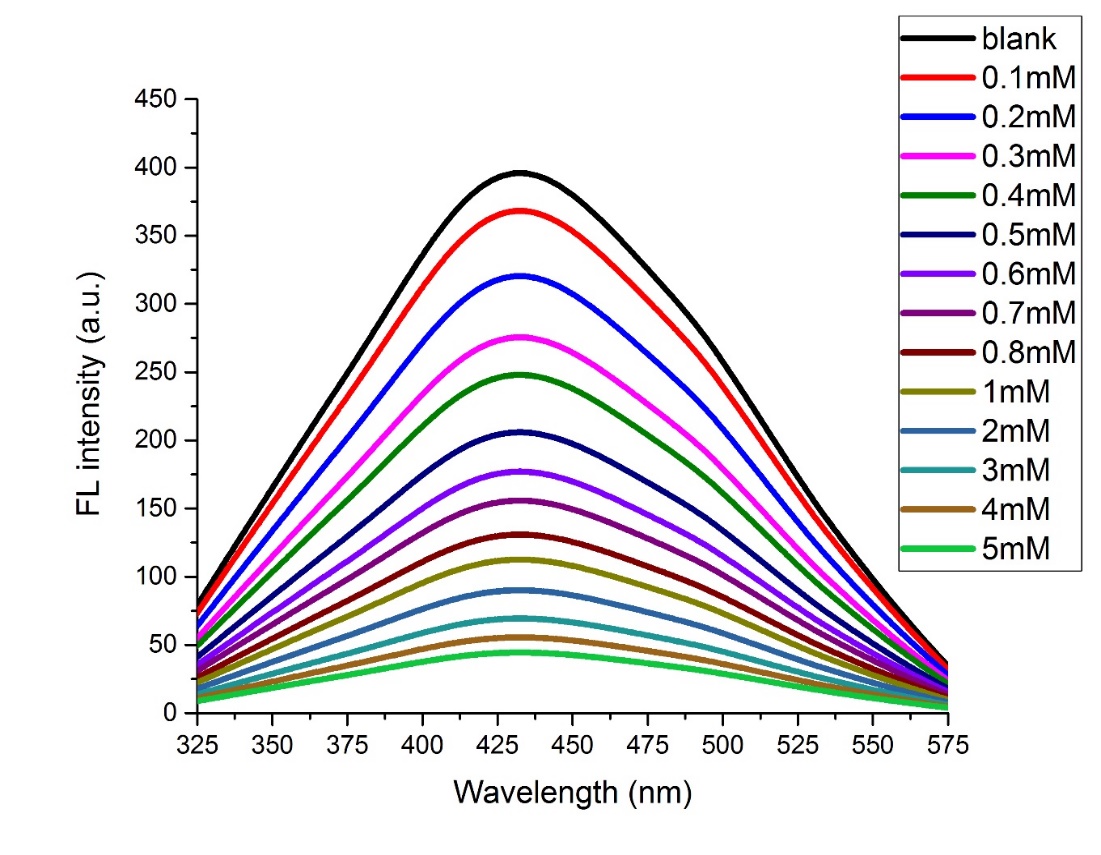


Figure **S5**. Fluorescence emission spectra of **TMU-50** dispersed in water solution at different concentrations of **1, 3-diheydroxybenzene,** excited at 310 nm.


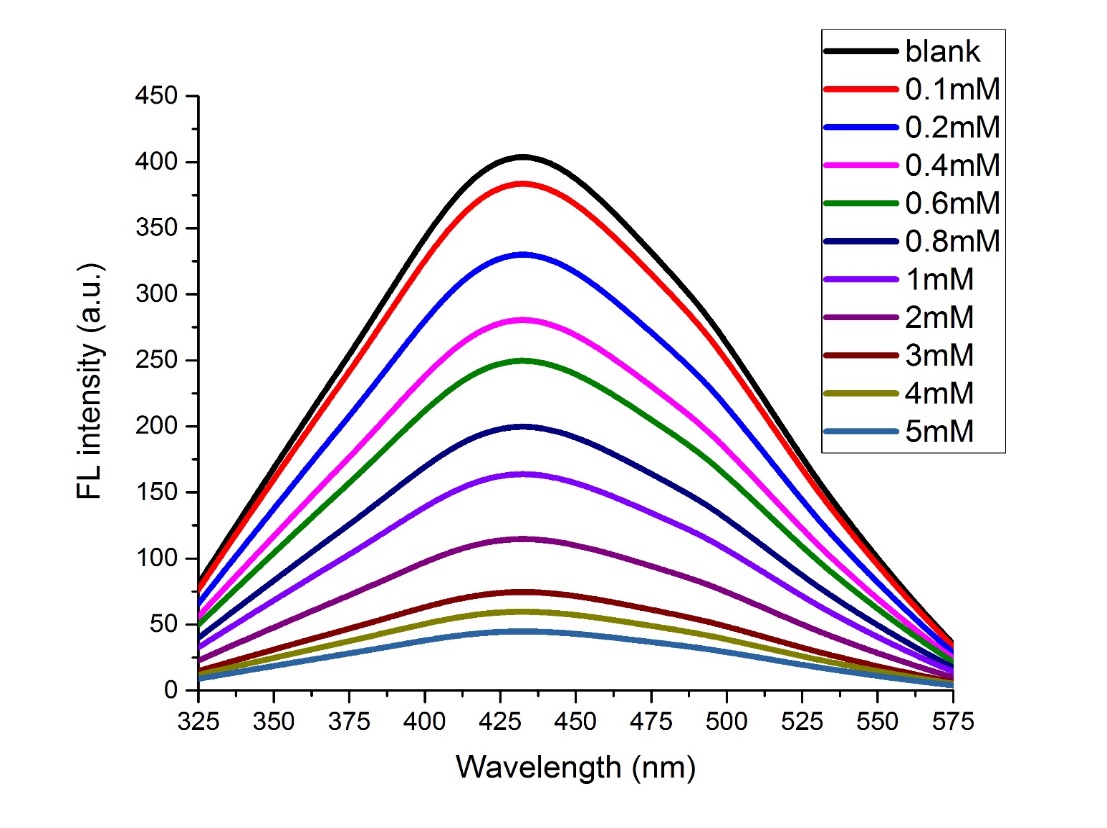


Figure **S6**. Fluorescence emission spectra of **TMU-50** dispersed in water solution at different concentrations of **phenol,** excited at 310 nm.


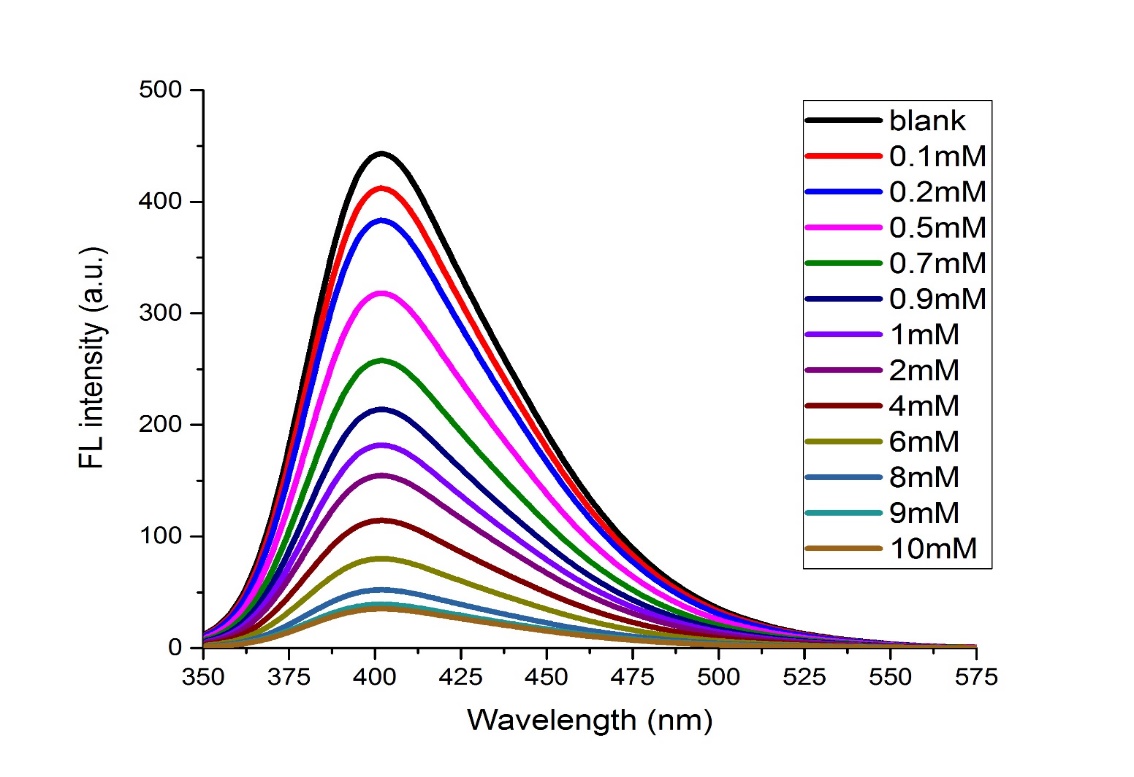


Figure **S7**. Fluorescence emission spectra of **TMU-51** dispersed in water solution at different concentrations of **4-Nitrophenol,** excited at 320 nm.


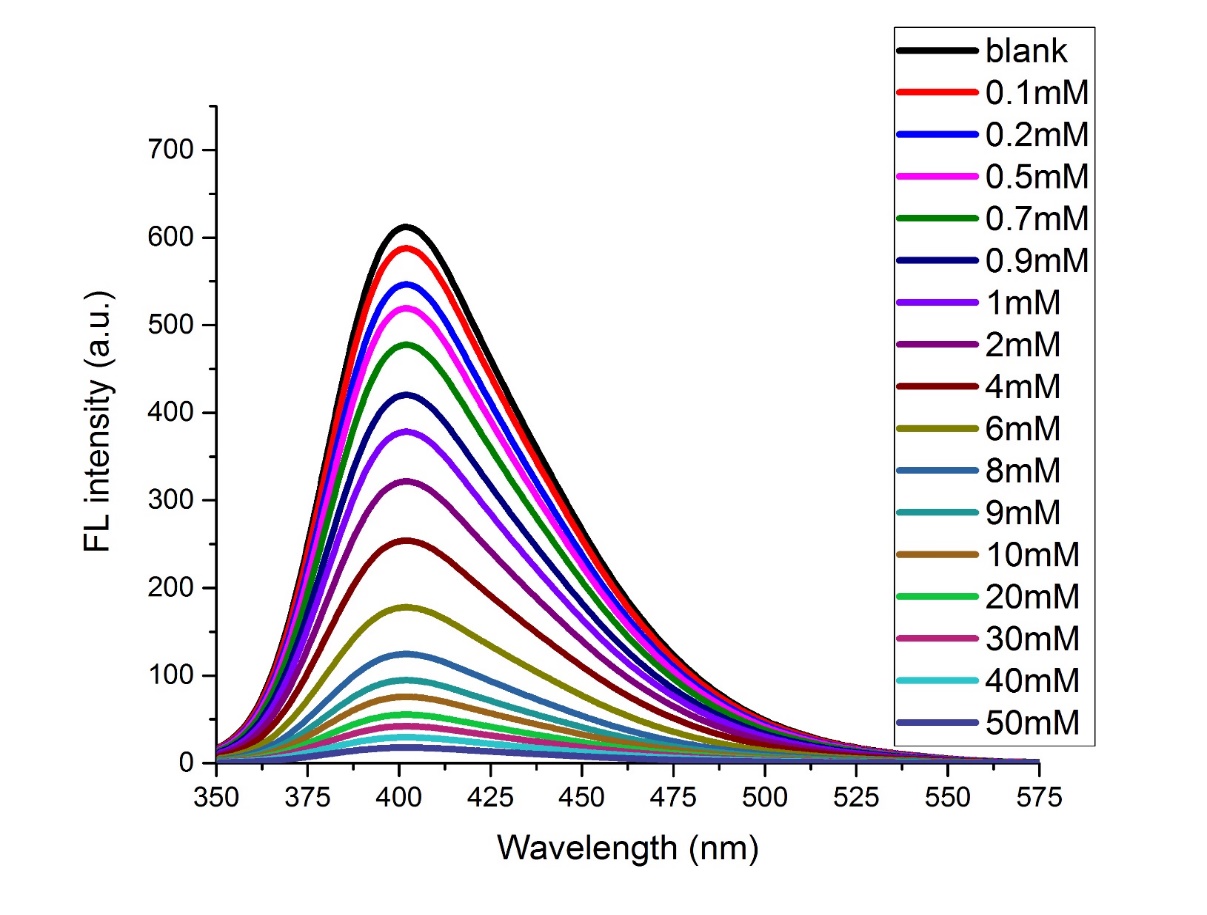


Figure **S8**. Fluorescence emission spectra of **TMU-51** dispersed in water solution at different concentrations of **2,4,6- trinitrophenol,** excited at 320 nm.


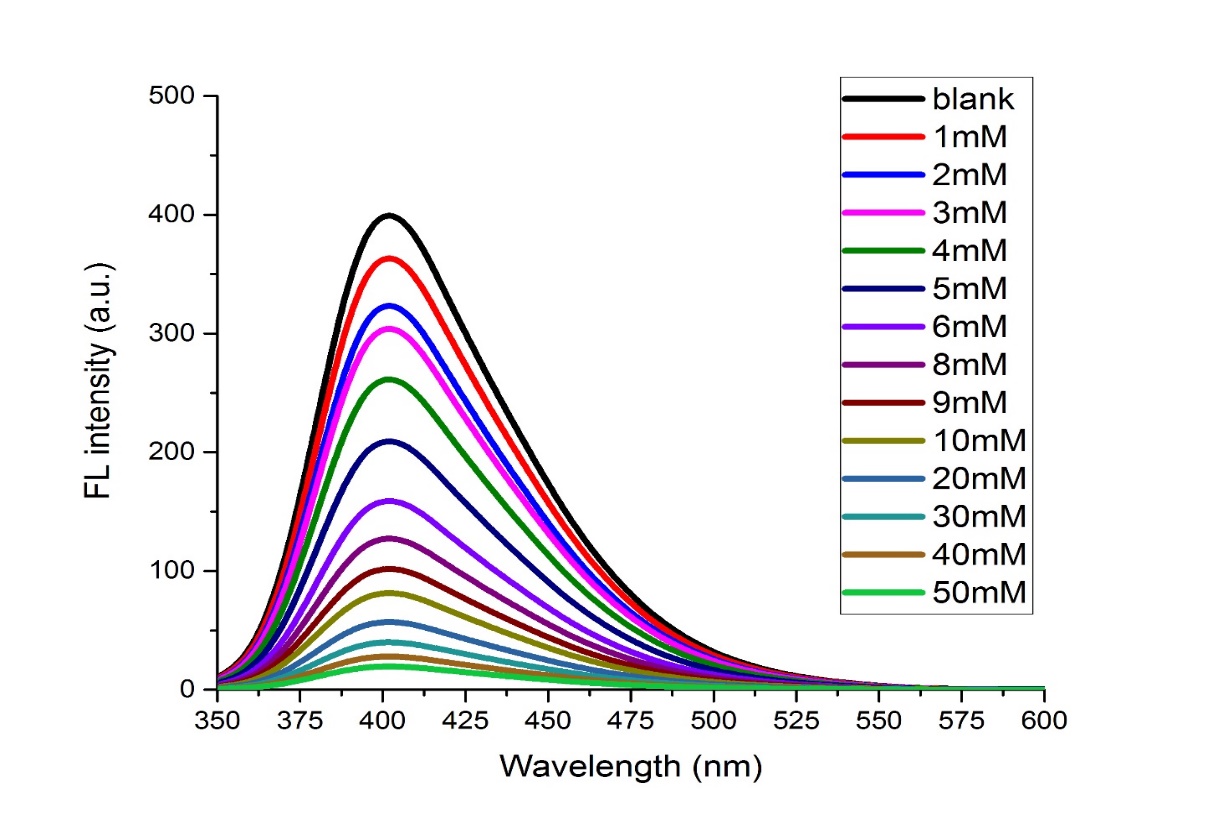


Figure **S9**. Fluorescence emission spectra of **TMU-51** dispersed in water solution at different concentrations of **4-Nitroaniline,** excited at 320 nm.


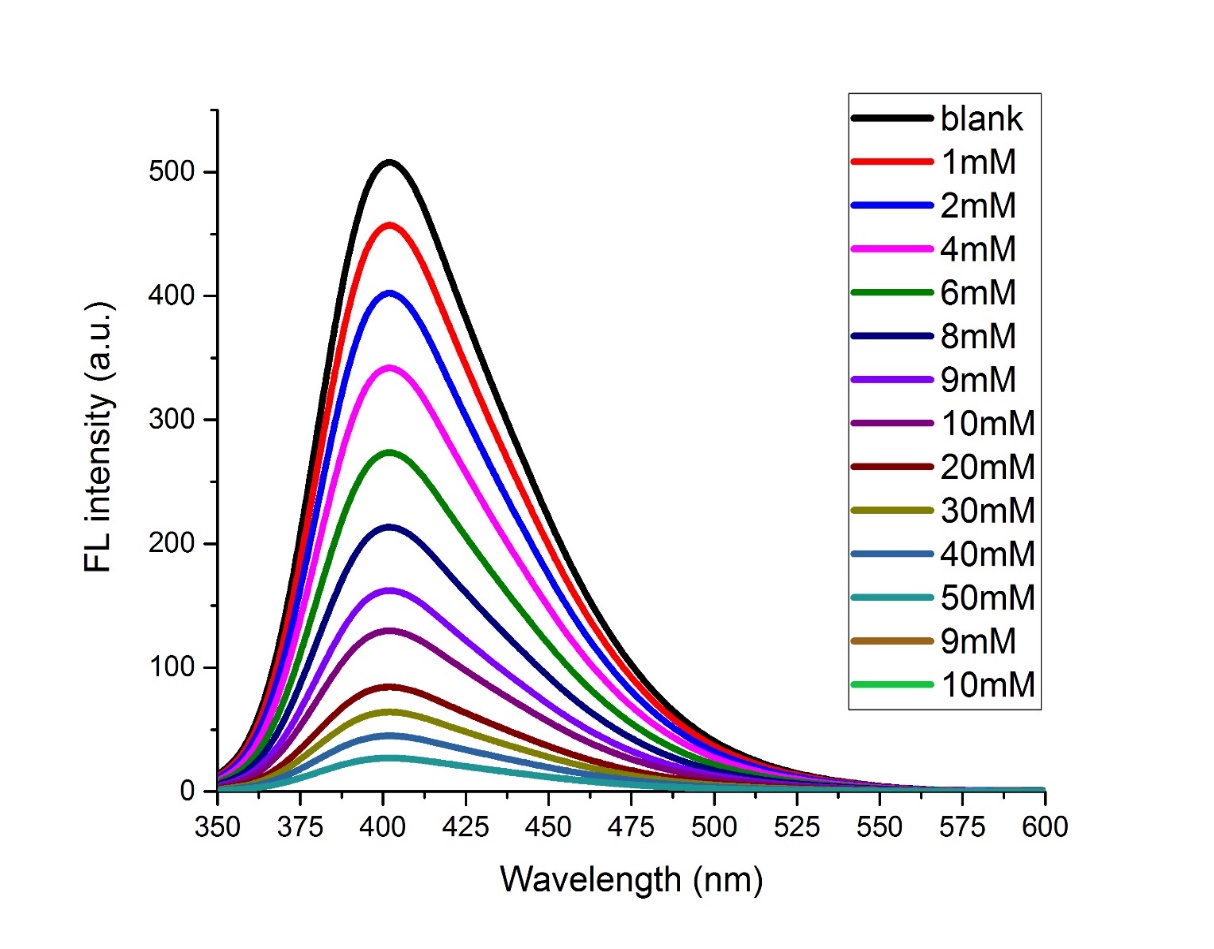


Figure **S10**. Fluorescence emission spectra of **TMU-51** dispersed in water solution at different concentrations of **4-methylphenol,** excited at 320 nm.


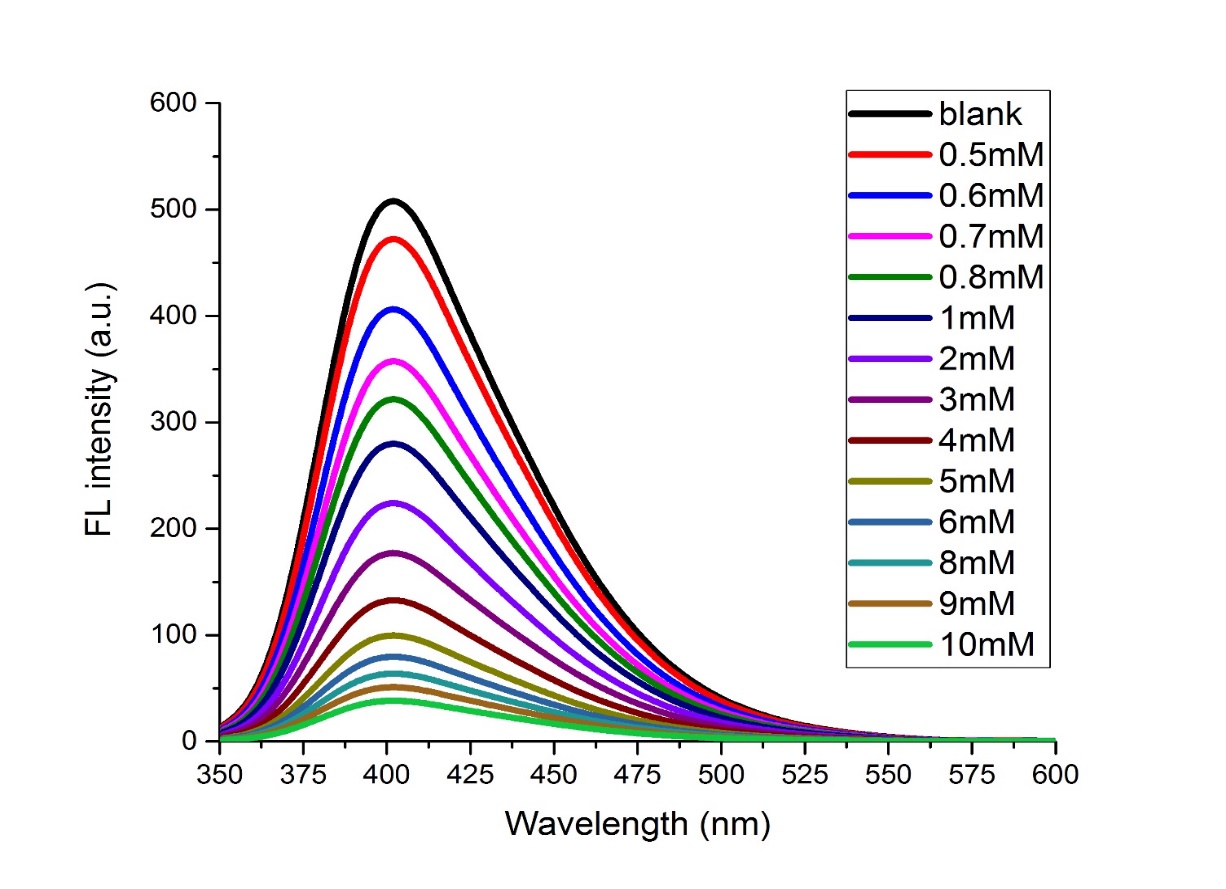


Figure **S11**. Fluorescence emission spectra of **TMU-51** dispersed in water solution at different concentrations of **1,3-dihedroxybenzene,** excited at 320 nm.


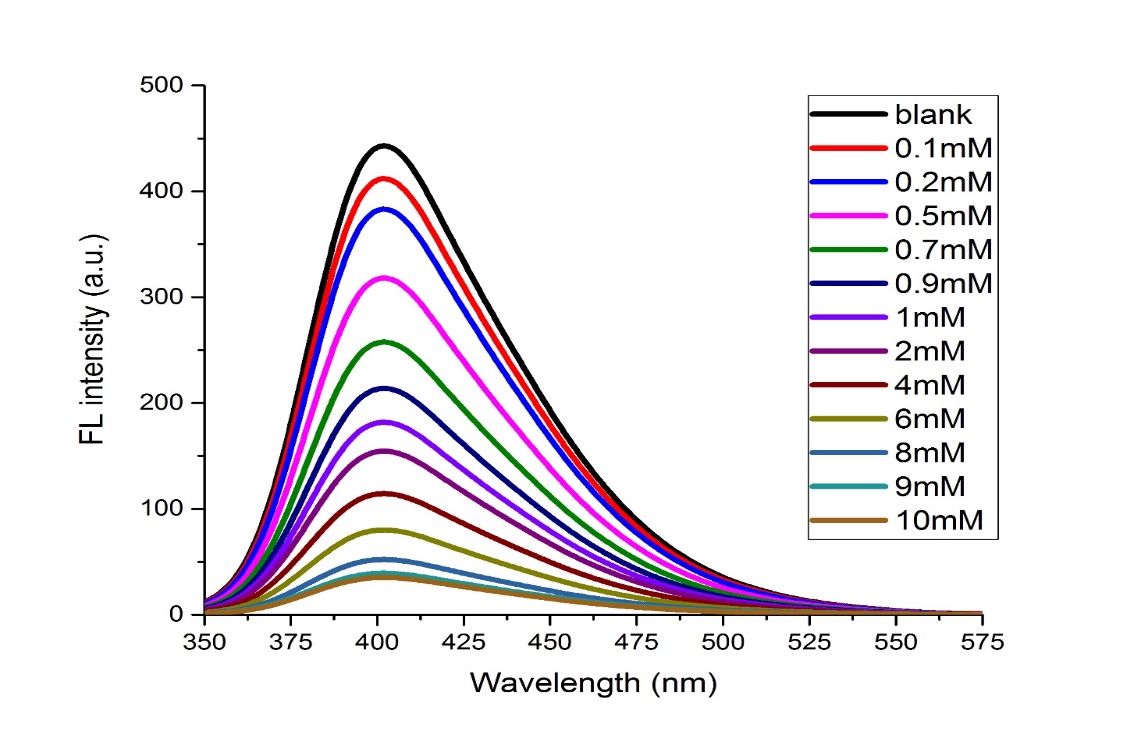


Figure **S12**. Fluorescence emission spectra of **TMU-51** dispersed in water solution at different concentrations of **phenol,** excited at 320 nm.


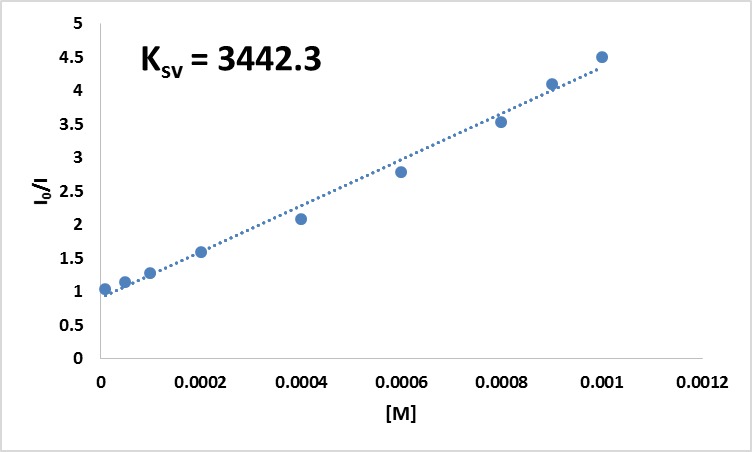


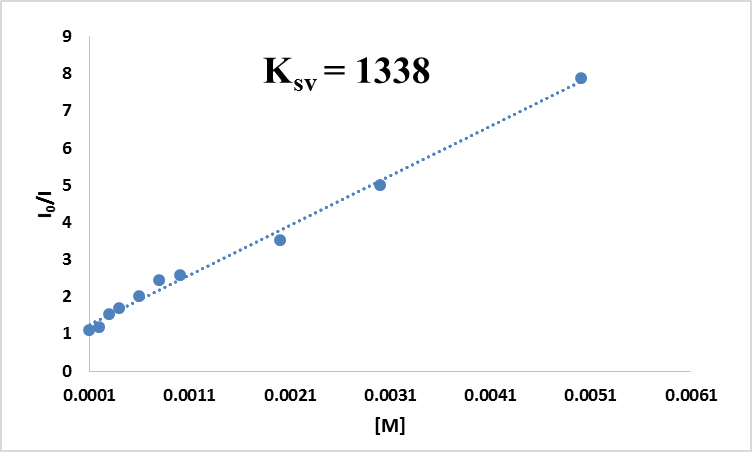


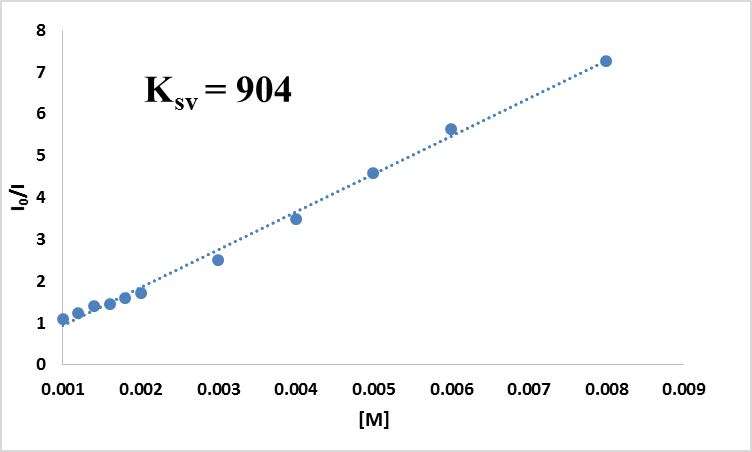


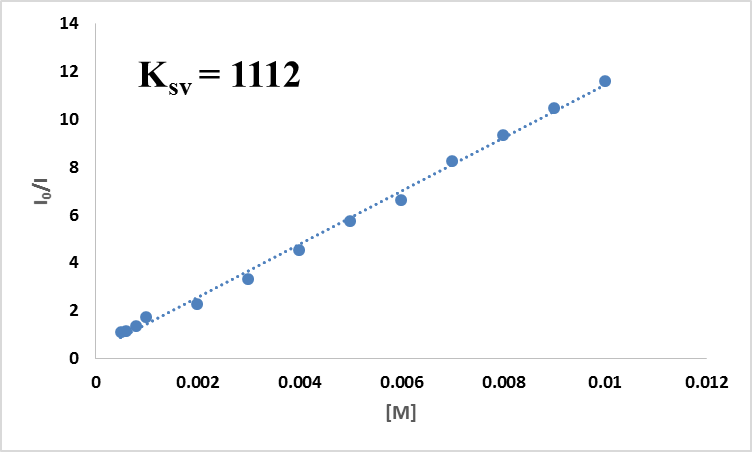


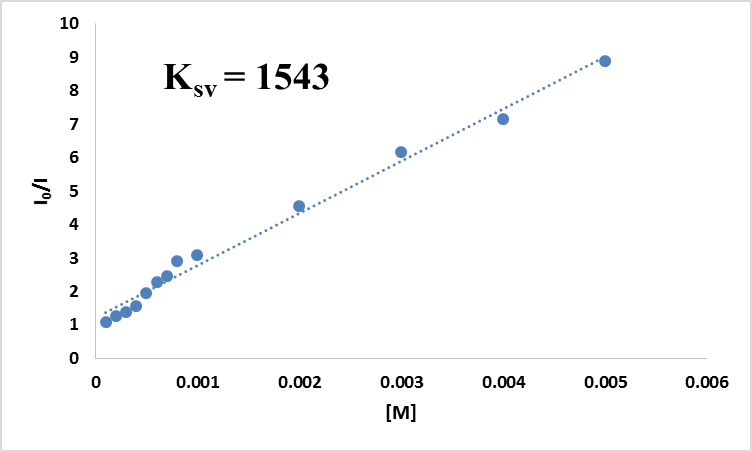


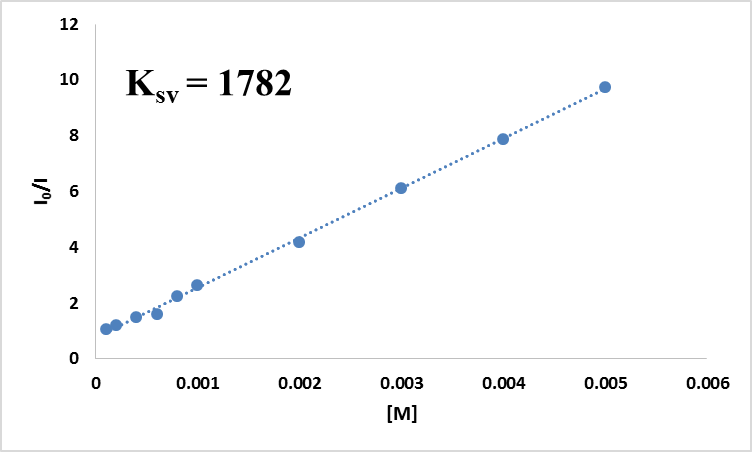


Figure **S13**. Stern–Volmer (SV) plots in the presence of 3 mg of **TMU-50** in different **4-Nitrophenol** (a) **2,4,6-trinitrophenol** (b) **Nitroaniline** (c) **4-methylphenol** (d) **1,3-dihedroxybenzene** (e) **phenol** (f) concentrations ([Q]) in water.


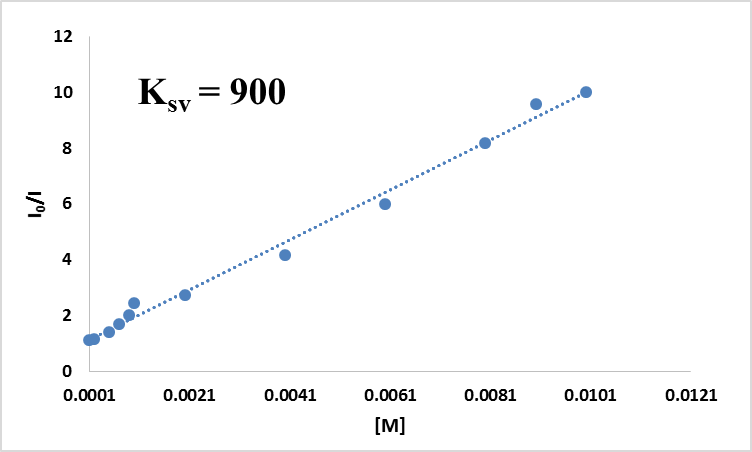


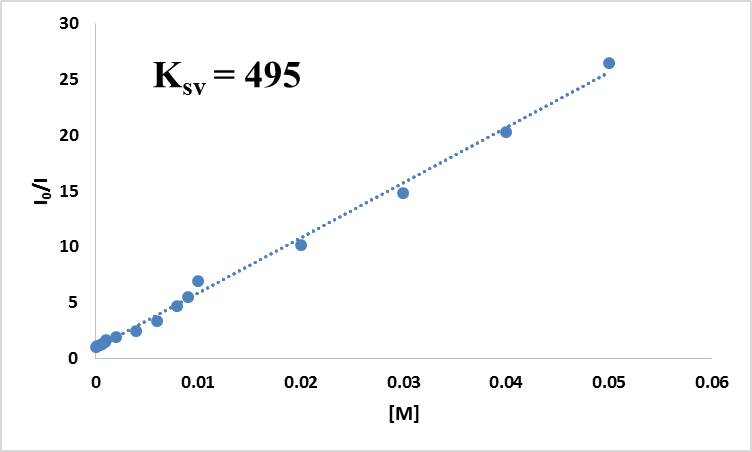


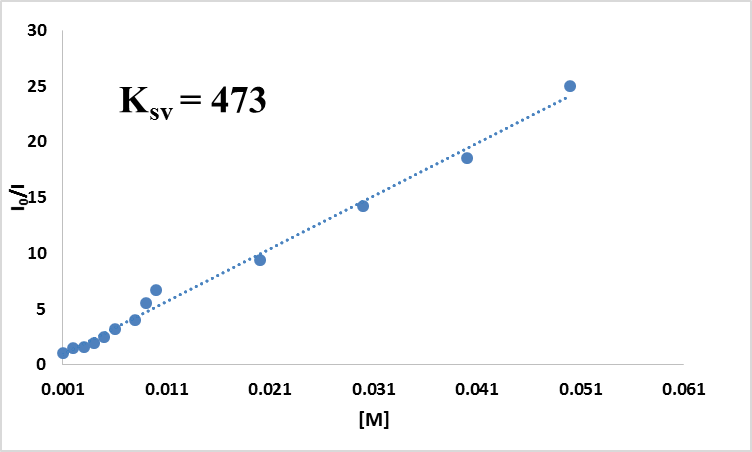


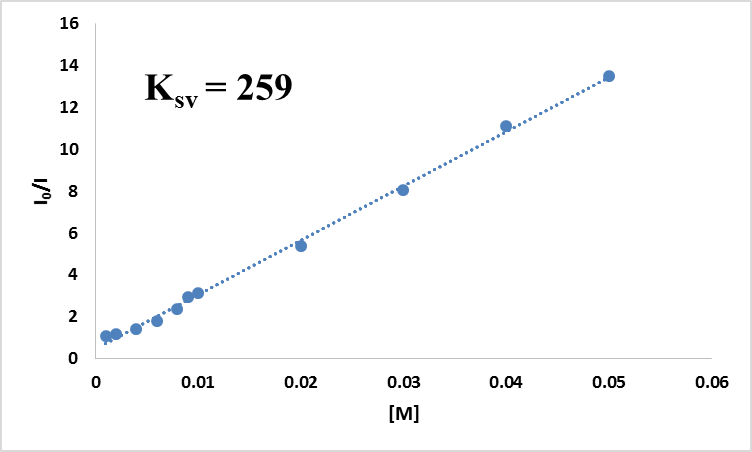


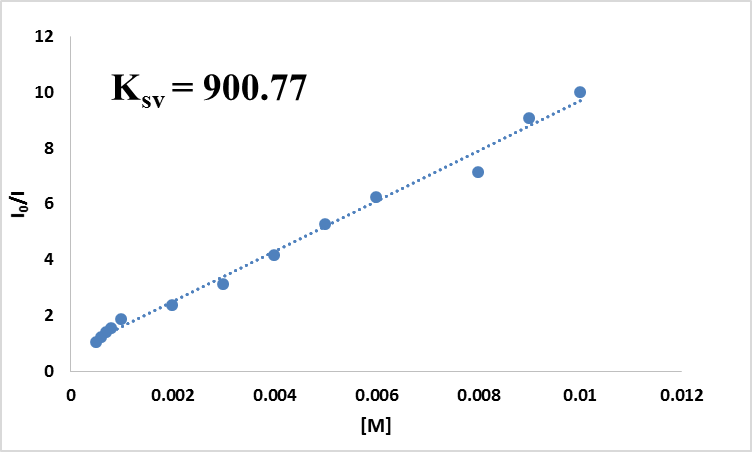


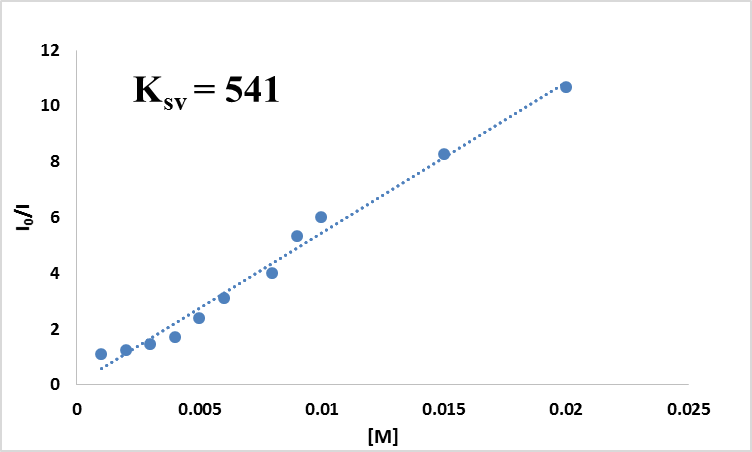


Figure **S14**. Stern–Volmer (SV) plots in the presence of 3 mg of **TMU-51** in different **4-Nitrophenol** (a) **2,4,6-trinitrophenol** (b) **Nitroaniline** (c) **4-methylphenol** (d) **1,3-dihedroxybenzene** (e) **phenol** (f) concentrations ([Q]) in water.
